# A flexible, high-throughput system for studying live mRNA translation with HiBiT technology

**DOI:** 10.1093/nar/gkaf496

**Published:** 2025-06-16

**Authors:** Camilla Ascanelli, Elsa Lawrence, Christopher A P Batho, Catherine H Wilson

**Affiliations:** Department of Pharmacology, University of Cambridge, 80 Tennis Court Road, Cambridge CB2 1PD, United Kingdom; Department of Pharmacology, University of Cambridge, 80 Tennis Court Road, Cambridge CB2 1PD, United Kingdom; Department of Pharmacology, University of Cambridge, 80 Tennis Court Road, Cambridge CB2 1PD, United Kingdom; Department of Pharmacology, University of Cambridge, 80 Tennis Court Road, Cambridge CB2 1PD, United Kingdom

## Abstract

HiBiT is an engineered luciferase’s 11-amino-acid component that can be introduced as a tag at either terminus of a protein of interest. When the LgBiT component and a substrate are present, HiBiT and LgBiT dimerize forming a functional luciferase. The HiBiT technology has been extensively used for high-throughput protein turnover studies in cells. Here, we have adapted the use of the HiBiT technology to quantify messenger RNA (mRNA) translation temporally *in vitro* in the rabbit reticulocyte system and *in cellulo* in HEK293 cells constitutively expressing LgBiT. The assay system can uniquely detect differences in cap, 5′UTR, modified nucleotide composition, coding sequence optimization and poly(A) length, and their effects on mRNA translation over time. Importantly, using these assays we established the optimal mRNA composition varied depending on the encoded protein of interest, highlighting the importance of screening methods tailored to the protein of interest, and not reliant on reporter proteins. Our findings demonstrated that HiBiT can be easily and readily adapted to monitor real-time mRNA translation in live cells and offers a novel and highly favourable method for the development of mRNA-based therapeutics.

## Graphical Abstract

**Graphical Abstract:**
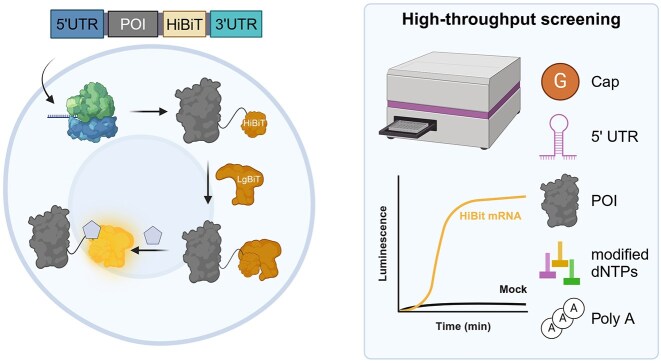

## Introduction

The use of messenger RNA (mRNA) therapeutics has expanded greatly since the roll-out of vaccines to combat the COVID-19 pandemic [1]. Aside from infectious diseases, mRNA therapies are being utilized for cancer vaccines and protein replacement therapeutics for conditions including cystic fibrosis, metabolic disorders, and cardiovascular disease [1–4]. The versatility of mRNA is exhibited by its ability to express any protein/s transiently, which, unlike protein/peptide therapeutics, do not rely on correct folding and modifications [4]. mRNA is unable to undergo genome integration [1, 3], providing a safer alternative to many DNA therapeutics. Overall, mRNA therapeutics demonstrate reduced toxicity when compared to other biologics [1, 3]. Furthermore, mRNA manufacturing is cell-free, enabling rapid, scalable, and low-cost production, making mRNA-based therapies attractive to drug developers.

Like mature mRNA, *in vitro* transcribed mRNA shares the same canonical features containing a 5′ cap, untranslated regions (UTRs), a coding sequence, and a poly(A) tail whilst also incorporating modified nucleotides to reduce immunogenicity [1, 5]. Multiple studies have investigated the effect these components play both individually and combinatorially on translation to achieve maximal protein expression [6, 7]. However, these studies have utilized highly engineered reporter proteins, including green fluorescent protein (GFP) [7], NanoLuc [8], and SARS-CoV-2 spike protein [8, 9], where translation is extremely efficient. As mRNA contains numerous structural elements and each therapeutic protein of interest (POI) is differentially regulated depending on cell type, identifying the optimum design for the therapeutic POI is essential.

Given the diverse effects different elements within an mRNA therapeutic have on translation and protein output, there is a need for assays that can assess these effects in mRNA research and development. Several ribosome-centric molecular assays have been adopted to assess translation dynamics of a given mRNA. For example, polysome profiling can differentiate low and high mRNA translation fractions through gradient centrifugation, offering insights into translational regulation through a single measurement [10]. Ribosome profiling (Ribo-seq) offers a more detailed, high-resolution snapshot of ribosome occupancy on mRNAs, providing a measure of translation rate, alternative initiation sites, and ribosome pausing [11]. HITS-CLIP and TRAP are similar sequencing-based techniques that offer details about translation of RNA-binding protein (RBP)-associated RNAs or ribosome-associated RNAs, respectively [12]. While these systems allow detailed investigation of a variety of mRNAs, they are not suitable for assessing elements within an mRNA for therapeutic development. Additionally, sample preparation is complex, highly specialized equipment and computational expertise are required, and they are not amenable to high-throughput or live cell assays.

Nascent chain immunoprecipitation [13] is a technique used for assessing translation efficiency that is based on a simple biochemical reporter assay. A plasmid expressing a FLAG-tagged protein is transfected and immunoprecipitated from elongated ribosomes with quantitative reverse transcription polymerase chain reaction (qRT-PCR), allowing quantification of eluted mRNA. This assay could be adapted for transfection of mRNA encoding FLAG-tagged POI, providing information on ribosome occupancy of the therapeutic RNA. Nonetheless, it remains time-consuming and does not allow for temporal resolution; thus, it is not compatible for high-throughput. SunTag is a system that allows visualization of nascent peptide chains from a single mRNA molecule [14]. The SunTag system relies on the interaction of a single-chain variable fragment (scFv) fused to GFP with up to 24 tandem repeats of the peptide epitope, which can be encoded in the mRNA upstream or downstream of the POI sequence. Unlike the aforementioned techniques, SunTag is able to measure active translation and localization using live cell imaging [15, 16], enabling comparisons between multiple POIs and providing single-cell resolution. However, the elements necessary for the system are bulky with one epitope alone double the length of HiBiT, thus potentially distorting the measured translation rate of the therapeutic.

A cell-free system that enables assessment of mRNA translation is the rabbit reticulocyte lysate (RRL) *in vitro* translation system. In the nuclease-treated lysate, all cell contents are preserved bar endogenous RNAs, which are degraded, thus maintaining all the necessary machinery for translation. The resultant low background of translation provides a convenient platform to measure translation efficiency directly from exogenously spiked mRNAs of interest [17, 18]. However, as this system is deprived of other mRNAs, it provides a simplified translational environment compared to cells, thus not recapitulating the complexities of mRNA regulation that affect translational output [19, 20]. Importantly, the tRNA landscape in rabbits [21] and the laborious detection methods required further limit the RRL system for high-throughput screening of potential therapeutic mRNAs. To adapt this popular assay to a flexible, high-throughput system, we have employed HiBiT technology to measure translation rates of synthetic mRNA.

HiBiT is a component of NanoLuc, an engineered luciferase, that can be introduced as an 11-amino-acid tag at either terminus of a POI. In the presence of the LgBiT component, and a substrate, HiBiT:LgBiT dimers form a functional luciferase that emits light in the visible spectra and can be detected using a luminometer [22, 23]. Since its discovery, HiBiT has been used to assess antiviral screening [24, 25], viral protein complex formation [26] and G protein-coupled receptor localization and function [27, 28]. However, its primary application has been in the study of protein dynamics, especially for measuring protein degradation as brought about by PROTACs [29], and the field of drug screening [30, 31]. HiBiT offers many advantages such as smaller size, less background, and lower risk of disrupting POI function compared to tags such as GFP and SunTag [14]. Unlike HA [32] and FLAG, which require antibody-mediated capture, HiBiT allows for higher throughput and live cell assays, making it attractive for investigating translation dynamics of therapeutic POI.

Here, we describe the use of HiBiT technology to quantify mRNA translation *in vitro* through endpoint measurements in the rabbit reticulocyte system and live *in cellulo* in HEK293 cells constitutively expressing LgBiT (Graphical abstract). For the first time, we introduce the HiBiT tag downstream of several POI sequences and produce HiBiT-encoding mRNA by *in vitro* transcription. The live cellular data were confirmed by endpoint studies in A549 cells and human embryonic stem cell-derived cardiomyocytes (hESC-CMs), demonstrating the versatility of the assay. Overall, the system described here can detect changes in translational and post-translational dynamics of mRNAs encoding different POIs and possessing various non-coding elements, making it well suited for high-throughput screening and optimization of mRNA-based therapeutics.

## Materials and methods

### Template plasmid construction for linear mRNA

Plasmids (pBluescript KS+, pBS hereon) for HiBiT-tagged POI mRNA were generated by Gibson Assembly (Gibson Assembly cloning kit, New England Biolabs (NEB), E5510S) or restriction digest cloning. Fragments consisting of digested pBS backbone, PCR product 5′UTR-POI-spacer-HiBiT, and PCR product HiBiT-3′UTR-AgeI were assembled by Gibson assembly according to the manufacturer’s instructions. Specifically, POI-spacer-HiBiT fragments were amplified from previously cloned POI-HiBiT fragments in pcDNA3 backbone. The 3′UTR fragment was amplified from a previously generated pBS-5′UTR_eGFP_3′UTR plasmid [33]. All PCRs were performed with Q5 High-Fidelity DNA Polymerase (NEB, M0491S) and digested with DpnI (NEB, R0176S). PCR and digest products were purified using Monarch DNA Gel Extraction and PCR and DNA Clean-Up Kits (NEB T1020S, T1030S).

To generate the pBS-eGFP-HiBiT-3′UTR construct lacking a 5′UTR (No 5′UTR), a PCR was performed using a forward primer lacking the 5′UTR but with the Kozak upstream of the ATG. The PCR product was cloned into the pBS backbone by restriction digest and ligation using T4 DNA ligase (NEB, M0202S). For the construct with a structured 5′UTR (5′UTR-hairpin), the RNAfold [34] webserver was used to predict the hairpin structure. The design consisted of a sequence forming a hairpin containing the Kozak sequence in the loop, with the start codon immediately downstream within the stem region of the hairpin. The fragment was synthesized by Thermo Fisher Gene Strings and cloned into the pBS backbone by restriction enzyme cloning.

For MycT58A mRNA optimization, the LinearDesign algorithm [9] was run with the MycT58A protein sequence using Python (version 2.7) and Clang (version 17.0.6). For Codon Adaptation Index (CAI) optimization, a Lambda value of 100 was chosen, and for Minimum Free Energy (MFE) optimization, a value of 0 was chosen. Myc T58A CAI and MFE were manufactured by GENEWIZ as gene synthesis constructs and cloned into pBS using restriction digest cloning. All constructs generated were confirmed by Sanger sequencing.

### Synthesis and purification of linear mRNA

For mRNA synthesis, 1 μg of plasmids pBS-5′UTR-POI-HiBiT-3′UTR were linearized by restriction enzyme digest and purified using the QIAGEN QIAquick PCR Purification Kit (Qiagen, 28104) according to the manufacturer’s instructions. Sequences for non-coding and coding elements used can be found in Supplementary Table 1. From the linearized plasmid, the template for mRNA synthesis was amplified by PCR with Platinum Taq DNA Polymerase High Fidelity (Invitrogen, 11304-011) according to the manufacturer’s programme specifications and custom primers (Supplementary Table 2). Following PCR clean-up, MEGAscript T7 Transcription Kit (Invitrogen, AM1333) was used to perform *in vitro* transcription for mRNA synthesis, with an incubation time of 16–18 h [33]. Several components of the mRNA synthesis were used in combination in this reaction: anti-reverse cap analog (ARCA) (NEB, S1411), CleanCap Reagent AG (Trilink, N-7113), pseudouridine-5′-triphosphate (Trilink, N-1019), N1-methylpseudouridine-5′-triphosphate (Trilink, N-1081), 5-methoxyuridine-5′-triphosphate (Trilink, N-1093), 5-methylcytidine-5′-triphosphate (Trilink, N-1014). Following the *in vitro* transcription, samples were treated with TURBO DNase (ThermoFisher, AM2238) for 15 min to degrade template DNA from the synthesis product. mRNA was purified using MEGAclear Transcription Clean-Up Kit (Invitrogen, AM1908) following the manufacturer’s instructions. Purified mRNA was treated with Antarctic phosphatase (NEB, M0289) for 1 h at 37°C followed by another purification round using MEGAclear. mRNA concentration was determined using a NanoDrop spectrophotometer (Thermo Scientific). Correct mRNA size was confirmed by bioanalyser and electrophoresis by running on 1% non-denaturing agarose gel. Purified mRNA was stored at −80°C.

### *In vitro* HiBiT translation assay in RRL

The *in vitro* translation assay was performed by adapting the protocol of Promega’s Flexi Rabbit Reticulocyte Lysate Kit (nuclease treated, L4540). Given the different sizes of the mRNA products, the comparison between constructs must be made with equal molecules of mRNA per reaction. For this, we recommend using 1.5 pmol of mRNA per reaction. First, mRNA and all components of the kit were thawed on ice. A reaction master mix was prepared and kept on ice as detailed in Table 1. For time course experiments, we recommend preparing the master mix to include as many mRNA samples as time points, plus one for pipetting errors, given the viscosity of the reticulocyte lysate mix.

**Table 1.** Reagents and volume of components of a RRL reaction

Component	mRNA sample (μl)	No RNA control (μl)
Flexi Rabbit Reticulocyte Lysate	8.25	8.25
Amino acid mixture minus leucine (1 mM)	0.25	0.25
Amino acid mixture minus methionine (1 mM)	0.25	0.25
KCl (2.5 M)	0.5	0.5
Ribonuclease inhibitor (Thermo Fisher Scientific, N8080119)	0.5	0.5
MgACO (25 mM)	0.5	0.5
DTT (100 mM)	0.25	0.25
RNA (0.75 pmol/μl)	2	0
Nuclease-Free H_2_O	0	2
Final V	12.5	12.5

Once prepared, the master mix was divided into separate tubes for each time point or mRNA condition, all kept on ice. For experiments aimed at comparing the translational efficiency of different mRNAs at a single time point, a master mix lacking mRNA was prepared and divided into separate tubes (10.5 μl/tube), to which 2 μl of mRNA were added separately to each tube. All tubes were then added to a heat block set at 30°C for the duration of the incubation and immediately placed back on ice. For time course experiments, appropriate tubes were removed according to the equivalent time point. A single time point of No RNA control with incubation of 120 min was run. Once all samples had cooled, they were stored at −80°C to ensure termination of the reaction. Translation reaction replicates were performed on different days, with each sample run in duplicate and measured on a plate-based luminometer. On ice, the resulting HiBiT protein was measured by placing 2.5 μl of each sample in a well of a white-walled 96-well plate (Nunc™ MicroWell™ 96-Well, Nunclon Delta-Treated, Flat-Bottom Microplate, 136101) in duplicate or triplicate. To minimize variability, all experimental replicates were processed in the same plate. To all wells, 50 μl of HiBiT buffer (LgBiT 1:200, Promega, N112A; Furimazine 1:100 Promega, N113A in nuclease-free water) were added. Samples and buffer were mixed briefly on a shaker maintaining samples on ice, then luminescence readings were acquired using a CLARIOstar Plus luminometer (530–540).

### Western blotting

For Western blotting of RRL samples, 1.6 μl of each replicate lysate were pooled to a final 5 μl volume. Sample buffer [50 mM Tris–HCl, pH 6.8, 2% sodium dodecyl sulfate (SDS), 0.1% bromophenol blue, 10% glycerol, 100 mM dithiothreitol added fresh] was added to bring to a final volume of 25 μl, then samples were heated to 80°C for 2 min for protein denaturation. Of the denatured samples, 4 μl were loaded on a 10%–15% acrylamide/bis gel (for better resolution of eGFP and eGFP-HiBiT sizes, a 15% polyacrylamide gel was used), and proteins were separated by electrophoresis at 150 V for 1.5–3 h, depending on the gel’s acrylamide percentage. Proteins were transferred to a pre-activated PVDF membrane in 1-Step Transfer buffer (Thermo Fisher, 84731) using a semi-dry Pierce G2 Fast Blotter (Thermo Fisher), which allows transfer at 25 V, 1.3 A in 15 min according to the manufacturer’s instructions. After transfer, membranes were blocked in 2% bovine serum albumin (BSA) in tris-buffered saline (TBS) with 0.1% Tween 20 for 1 h at room temperature and incubated overnight in rabbit anti-eGFP (1:1000, abcam, ab290), mouse anti-GFP (1:1000, Roche, 11814460001) and mouse anti-GAPDH (1:5000, Proteintech, 60004-1-Ig). Following incubation with primary antibodies, membranes were washed with TBS with 0.1% Tween 20 three times, with each wash lasting 5 min, followed by 1-h incubation with secondary antibody solution (0.01% SDS, 20% fetal bovine serum (FBS), TBS with 0.1% Tween 20) containing fluorescent secondary antibodies IRDye 680RD Goat anti-Rabbit IgG Secondary Antibody (1:20 000, Li-COR Biosciences 926-68071) and IRDye 800CW Goat anti-Mouse IgG Secondary Antibody (1:10 000, Li-COR Biosciences 926-32210). Following incubation with secondary antibodies, membranes were washed with TBS with 0.1% Tween 20 three times and imaged using an Odyssey Li-COR Imaging System (Li-COR Biosciences). Following imaging of the fluorescent signal, membranes were incubated for 1 h with LgBiT buffer (0.5% LgBiT in TBS with 0.1% Tween 20). In the last 5 min of incubation in LgBiT buffer, Furimazine substrate was added to the buffer at a 500-fold dilution. The membranes were imaged again for bioluminescence recording of HiBiT:LgBiT signal using the same imaging system. The resulting images were analysed on Image Studio Lite (Li-COR Biosciences) by measuring the average fluorescence intensity of a region of interest (ROI) around the respective band and subtracting the intensity reading of an ROI of the same size in the No RNA control. The background-corrected signal was then normalized to GAPDH loading control.

### Cell culture

HEK293 LgBiT and HEK293 Myc-HiBiT LgBiT cells (gift from Itzhaki lab) were cultured in Dulbecco’s Modified Eagle’s Medium (DMEM, Thermo Fisher, 41966052) supplemented with l-glutamine (Thermo Fisher, 25030-024) and 10% FBS (Sigma, F7524). A549 cells (gift from Downward lab) were cultured in Roswell Park Memorial Institute medium (RPMI 1640, ThermoFisher, 231875091) supplemented with l-glutamine and 10% FBS (Sigma, F7524). Human hESC-CMs were differentiated from hESCs exactly as described previously [33]. Briefly, H9/WA09 hESCs were cultured in TeSR-E8 (StemCell Technologies, 05990) with daily media change. In preparation for the differentiation, hESCs were dissociated using TrypLE Express (Thermo Fisher, 12604021) and seeded at 1 million cells per well on Matrigel-coated six-well plates. Cardiomyocyte purity was measured using flow cytometry and a Troponin T antibody (Miltenyi, 130-120-403) to ensure it was > 70%. All cells were maintained at 37°C, 5% CO_2_, and routinely tested for *Mycoplasma*.

### Live *in cellulo* HiBiT translation assay

For live-cell experiments of mRNA translation dynamics, HEK293 LgBiT cells were reverse-transfected with 1.2 pmol of mRNA with Lipofectamine RNAiMAX Transfection Reagent according to the manufacturer’s instructions and plated on clear bottom, white-walled plates (Greiner Bio-One CELLSTAR 96-well, White, Cell Culture-Treated, Flat-Bottom Microplate, 655098) at 40 000 cells/well in Leibovitz’s L-15 Medium no phenol red (Thermo Fisher, 21083-027), which supports cell growth in environments without CO_2_ equilibration, supplemented with 10% FBS and substrate Nano-Glo Endurazine Live Cell Substrate (Promega, N2570, 1:200). Immediately post-transfection, cells were placed in a CLARIOstar Plus luminometer set to 37°C, where recordings were taken every 12 min for 18 h (530–540). Negative controls of mock transfections lacking RNA were performed. For live cycloheximide-chase experiments, HEK293 Myc-HiBiT LgBiT cells were plated in triplicate as above at 100 000 cells/well with cycloheximide (Sigma–Aldrich, 01810-1G, Cf = 100 μg/ml) or vehicle control dimethyl sulfoxide (Sigma–Aldrich, 276855, DMSO, Cf = 0.5%). Luminescence readout was measured as above.

### *In cellulo* HiBiT endpoint lytic assay

Cells were reverse-transfected with 1.2 pmol of mRNA with Lipofectamine RNAiMAX Transfection Reagent according to the manufacturer’s instructions and plated on clear bottom, white-walled plates (Greiner Bio-One CELLSTAR 96-well, White, Cell Culture-Treated, Flat-Bottom Microplate, 655098) at 50 000 cells/well in appropriate media (RPMI for A549, CDM-BSA for hESC-CMs) and incubated at 37°C. After 4 h, cells were washed with Dulbecco's Phosphate Buffered Saline (DPBS) (Sigma, D8537) and lysed with lytic buffer composed of 0.05% Triton X-100 (VWR, 0694), LgBiT (1:200, Promega, N112A), and Furimazine (1:100 Promega, N113A) in DPBS. The plate was protected from light and placed on a shaker for 5 min. Then, the plate was placed in a CLARIOstar Plus luminometer where luminescence measurements were taken (530–540). Negative controls of mock transfections lacking RNA were included.

### Quantitative reverse transcription PCR

To measure mRNA transfection, stability, and immunogenic response, qRT-PCR was performed on cells after transfection. Briefly, cells were reverse-transfected with 5 pmol of mRNA with Lipofectamine RNAiMAX Transfection Reagent according to the manufacturer’s instructions and plated on a clear 24-well tissue culture plate (Corning, 3526) at 160 000 cells/well in DMEM (Thermo Fisher, 41966052) supplemented with l-glutamine (Thermo Fisher, 25030-024) and 10% FBS (Sigma, F7524). At 4 and 18 h post-transfection, medium was removed, and wells were washed with DPBS to prevent carryover of mRNA that was not transfected into cells. Cells were then dissociated using Trypsin-EDTA (Sigma, T4174) and collected for centrifugation. The cell pellet was washed thoroughly with DPBS and stored at −80°C until ready for RNA extraction. Total RNA was isolated using NucleoSpin RNA Mini kit (Macherey-Nagel, 74095550) according to the manufacturer’s instructions. Once isolated, NanoDrop Lite spectrophotometer (Thermo Fisher Scientific) was used to quantify and assess the purity of RNA. RNA was reverse transcribed using the High-Capacity cDNA Reverse Transcription kit (random primers) (Thermo Fisher Scientific, 4368814) according to the manufacturer’s instructions. The qRT-PCR reactions were performed by the CFX Opus 384 (Bio-Rad) using 5 μl Fast SYBR Green Master Mix (Applied Biosystems, 4385612), 0.5 μl of each oligonucleotide primer, and 1 μl complementary DNA (cDNA) to a final volume of 10 μl, run in duplicate technical replicates. The delta–delta Ct method [35] was used to determine gene expression changes using GAPDH as a housekeeper gene. Primers for target genes (Supplementary Table 2) were synthesized by Merck and were used at a final concentration of 250 nM.

### Statistics

Statistical analyses were performed on normalized data using GraphPad Prism v10.1.1 (GraphPad Software, Inc., San Diego, CA, USA) as indicated with *P* ≤ 0.05 considered statistically significant.

For *in vitro* HiBiT translation experiments, luminescence recordings for each sample were normalized to the average of the respective No RNA control. Given the novelty of this assay, the *Z*′ (or *Z*-factor), a statistical measurement of the robustness of assays, was calculated by comparing the signals from the minimal and maximal signals recorded in the assays [36]. For the RRL HiBiT assay, No RNA and eGFP-HiBiT 40A samples, respectively, were used as minimum and maximal signal, resulting in a *Z*′ = 0.8, indicating an optimal dynamic range.

For live, *in cellulo* HiBiT translational assays, each condition’s signal was normalized to its first time point, as the signal at the beginning is background level for all conditions, to generate fold changes in relative luminescence units (RLUs). Once more, the *Z*′ was calculated, using the peak and end values (4- and 18-h time points, as plotted in Fig. 5C) for eGFP-HiBiT with poly(A) of 40A against No RNA control, which resulted in a *Z*′ = 0.8.

Statistical analysis of *in cellulo* data normalized to time 0 was performed at 4 or 18 h by one-way analysis of variance (ANOVA) with multiple comparisons made to the reference HiBiT mRNA. Furthermore, to assess the contribution of coding and non-coding elements to the translation of POI-HiBiT at 4 and 18 h and measure dynamics of translation and protein turnover, the area under the curve (AUC) for all live cell experiments was calculated using GraphPad Prism for curves from time 0 to 4 h and for the full 18 h.

AUC values per biological replicate were normalized to the average of the reference construct, yielding a measure of fold change in AUC, and analysed by two-way ANOVA with matched values to reflect continuous data. For comparisons of delta–delta Ct values at 4 and 18 h from qRT-PCR, we applied a two-way ANOVA.

## Results

### The addition of HiBiT does not hinder the translation of eGFP *in vitro* and offers a high-throughput system for monitoring translational dynamics

**Figure 1.**
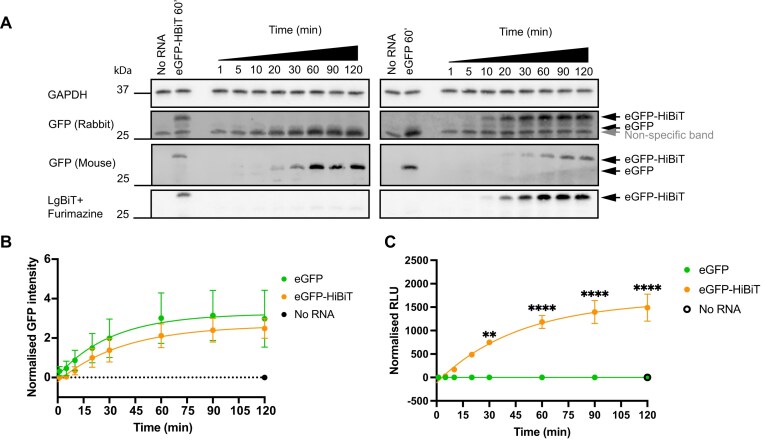
The addition of the HiBiT tag allows the detection of POI-specific translational dynamics profiles. (**A**) Translation dynamics of eGFP and eGFP-HiBiT in the RRL system by western blotting. Due to the presence of a non-specific band (grey arrow) when probing with a rabbit antibody, the specificity of the signal was confirmed with an antibody raised in a different species (mouse). Quantification of relative protein abundance in panel (**B**) normalized to GAPDH, eGFP (green), or eGFP-HiBiT (orange). (**C**) Translation dynamics of mRNA encoding eGFP (green) or eGFP-HiBiT (orange) measured by luminometer, RLUs of all samples were normalized to No RNA sample. Mean and standard error of the mean (SEM) of three biological replicates are plotted, and two-way ANOVA was conducted to compare eGFP and eGFP-HiBiT signal at each time point. **P< 0.005, ****P< 0.0001.

To determine whether the addition of a tag encoding HiBiT at the 3′ end of eGFP (fused to a nuclear localization signal—NLS) alters the translation dynamics of the fluorophore, two mRNAs expressing eGFP and eGFP-HiBiT were synthesized. These mRNAs possess identical cap, 5′- and 3′-UTR, and poly(A) tail lengths (120 adenosines) and differ only in the presence of a serine and glycine linker and the HiBiT coding sequence. We employed the cell-free *in vitro* translation assay RRL system to evaluate the rate of translation of the mRNAs over a time course (1, 5, 10, 20, 30, 60, 90, and 120 min). Western blotting antibody-based detection of proteins was performed, followed by direct incubation with LgBiT and substrate Furimazine to detect HiBiT-tagged eGFP by bioluminescence (Fig. 1A). Both methods provided an accurate and sensitive measurement of HiBiT-tagged protein; however, direct detection of eGFP-HiBiT does not rely on an antibody against a POI and confirmed that the higher molecular weight band detected by the GFP antibody was eGFP-HiBiT. Quantification of the efficiency of translation confirmed that the addition of HiBiT did not affect the translation dynamics of eGFP (Fig. 1B).

Despite the method sensitivity, western blotting results in high variability (large error bars), is low-throughput and time-consuming; therefore, a plate-based assay was developed. For this, RRL samples were incubated with LgBiT and substrate Furimazine for 5 min before luminescence readings were acquired. The assay produced less variable results and reproduced similar translation dynamics seen by western blotting (Fig. 1C). The assay specifically detected the HiBiT:LgBiT-induced luminescence as eGFP samples lacking the HiBiT tag did not produce a bioluminescence reading. Importantly, since the assay is compatible with a multi-well format, it allows for triplicate readings, and testing of multiple experimental replicates or samples in one plate, making this assay optimal for high-throughput work.

HiBiT-encoding mRNA can be used to study the contribution of the non-coding and coding elements of mRNA to its translation *in vitro* and *in cellulo*:

### CAP and 5′ UTR

**Figure 2.**
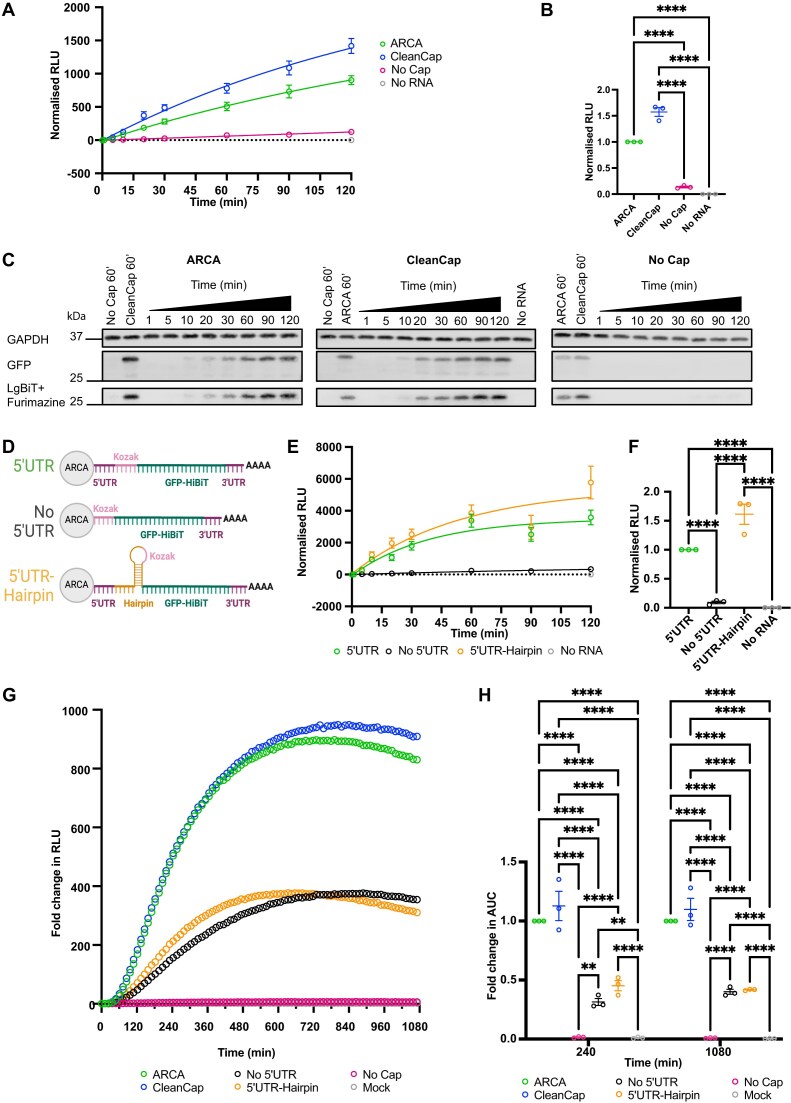
HiBiT-encoding mRNA can detect alterations to the 5′UTR. (**A**) Translation dynamics of mRNA capped using ARCA (green), CleanCap (blue), or uncapped mRNA (magenta) encoding eGFP-HiBiT were compared in the RRL system; RLU was normalized to No RNA control. (**B**) Scatter plot depicting RLU of three biological replicates at 120 min normalized to ARCA sample. Mean and SEM of three biological replicates are plotted, and ordinary one-way ANOVA was conducted. (**C**) Western blot of pooled RRL samples with GAPDH as a loading control, with eGFP-HiBiT protein detected with an anti-GFP antibody and LgBiT and Furimazine. (**D**) Schematic representation of the mRNA structures with a 5′UTR, No 5′UTR, and 5′UTR-hairpin. (**E**) mRNA translation dynamics of 5′UTR, No 5′UTR, and 5′UTR-hairpin constructs were compared in the RRL. RLU was normalized to No RNA control. (**F**) Scatter plot depicting RLU of three biological replicates at 120 min normalized to 5′UTR mRNA. Mean and SEM, and ordinary one-way ANOVA. (**G**) Fold change in RLU from time 0 of HEK293 LgBiT cells transfected with eGFP-HiBiT mRNA with: ARCA-capped (green), CleanCap-capped (blue), no Cap (magenta), No 5′UTR (black), 5′UTR-hairpin (orange), or mock (grey). Mean fold change RLU per time point is shown for the complete time course. (**H**) Mean and SEM of fold change in AUC of curves from 0 to 4 h or to 18 h of three biological replicates were obtained by normalizing to reference sample ARCA and compared by two-way ANOVA with matched values. *P< 0.05, **P< 0.005, 0.0001 < ***P< 0.0005, ****P< 0.0001. All data are from three biological replicates.

To further validate the sensitivity of the HiBiT assay, we evaluated the contribution of cap-dependent translation by comparing eGFP-HiBiT mRNA with no cap, ARCA, and CleanCap AG in the RRL system and measured on a plate-based luminometer (Fig. 2A–C). We determined that the cap is crucial for efficient protein translation, and the absence of cap significantly (*P*< 0.0001) reduced the detected luminescent signal (Fig. 2A and B) and protein level (Fig. 2C). No significant difference was measured between ARCA and CleanCap in this *in vitro* system (Fig. 2B).

To further challenge the HiBiT assay system, the properties of the 5′UTR were altered by designing two new mRNAs, one without a 5′UTR (No 5′UTR), and one which contained an 18 bp complementary region, resulting in a hairpin structure (5′UTR-hairpin) engineered immediately adjacent to the 5′UTR, concealing the start codon within the stem (Fig. 2D). The thermal stability and free energy of these sequences were estimated using the RNAfold [34]. The 5′UTR is predicted to have an MFE of −7.7 kcal/mol and thermodynamic ensemble free energy of −9.04 kcal/mol. The 5′UTR-hairpin is predicted to have an MFE of −43.4 kcal/mol and thermodynamic ensemble free energy of −46.89 kcal/mol. The translational dynamics of these constructs were measured with the HiBiT RRL system. No HiBiT:LgBiT signal could be detected in the absence of the 5′UTR, indicating little to no protein was synthesized over the course of the experiment, while both mRNAs with intact 5′UTRs generated a similar translation profile (Fig. 2E). When comparing the luminescent signal at 120 min, no significant difference was detected between mRNA with 5′UTR and 5′UTR-hairpin, but both constructs produced significantly higher signal than No 5′UTR and No RNA control (*P*< 0.0001, Fig. 2F).

To detect translation in live cells, we harnessed the HiBiT system to express HiBiT-tagged proteins in HEK293 cells constitutively expressing LgBiT. The experimental method allows the live recording of the dynamics of translation and post-translational processing of the transfected mRNA and resulting HiBiT-tagged protein. HEK293 LgBiT cells were reverse-transfected with the eGFP-HiBiT mRNAs (ARCA, CleanCap, No Cap, No 5′UTR and 5′UTR-hairpin) and treated with a stable substrate for the luciferase dimer, Endurazine. The mRNA with No Cap significantly abolished translation (*P*< 0.0001), whilst mRNA capped with ARCA and CleanCap resulted in a rapid increase in protein production, reaching plateau at 720 min (Fig. 2G). Contrary to the RRL, the presence of a structured element adjacent to the 5′UTR (5′UTR-hairpin) in addition to the absence of the 5′UTR (No 5′UTR) resulted in significantly slower translational dynamics compared to ARCA-capped mRNA with the 5′UTR (Fig. 2G). In cells, translation of mRNA with No Cap was completely abolished at both 4 and 18 h (Supplementary Fig. 1A and B). To investigate translation dynamics of these mRNAs, we calculated fold-change AUC values of curves from time 0 to 4 h or 18 h, normalized to the ARCA control. The analysis revealed that protein output from uncapped mRNA was initially low and reduced further over the course of 18 h, with an 84.9%-fold change AUC over 4 h and 91.1% overall decrease over 18 h. Removing the 5′UTR resulted in a fold-change decrease in AUC of 68.8% and 60%, over 4 and 18 h, respectively (No 5′UTR, Fig. 2G and H). Signal from the 5′UTR-hairpin sample also demonstrated reduced translational dynamics compared to ARCA capped mRNA containing an unstructured 5′UTR, with the fold change AUC dropping consistently by 54.9% and 58.3% reduction over 4 and 18 h, respectively (Fig. 2G and H).

To assess mRNA levels in cells that could affect signal output, we collected HEK LgBiT cells at 4 and 18 h post-transfection, isolated total cellular mRNA and performed qRT-PCR analysis. Results reveal no significant difference in levels of ARCA-, CleanCap-, and uncapped mRNA at 4 or 18 h, despite the big reduction in luminescence output for uncapped mRNA. In addition, there was no significant difference in levels of mRNA with 5′UTR (ARCA), No 5′UTR, and 5′UTR-hairpin, demonstrating that the reduction in luminescence in cell assays is not due to lack of transfection or degradation of the mRNA, but instead due to translational efficiency (Supplementary Fig. 1C). These results highlight the ability of this novel sensitive cellular assay to measure changes in the translation efficiency of mRNAs with differing non-coding elements at the 5′ end.

### Coding sequence

**Figure 3.**
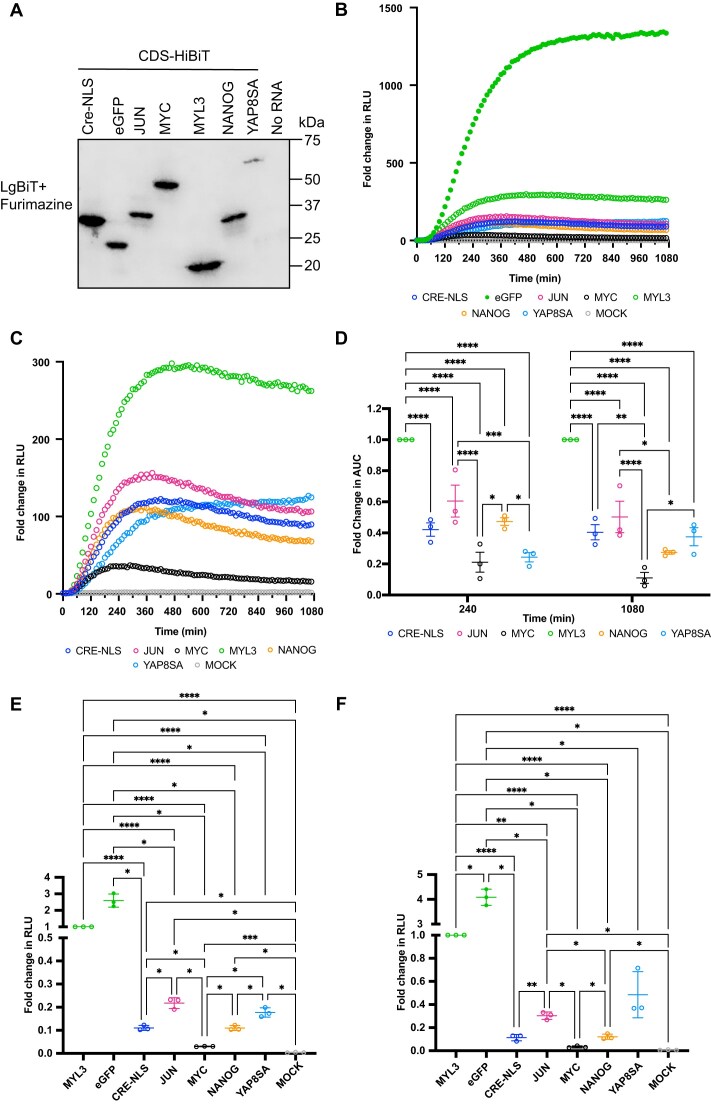
HiBiT-encoding mRNA detects changes in translational dynamics of different coding sequences. (**A**) RRL reactions were carried out for 120 min with mRNA encoding Cre-NLS-HiBiT, eGFP-HiBiT, Jun-HiBiT, Myc-HiBiT, MYL3-HiBiT, NANOG-HiBiT, YAP8SA-HiBiT, or No RNA. Resulting proteins were processed for western blotting and detected by LgBiT and Furimazine. Live cellular kinetic assay in HEK293 LgBiT cells transfected with eGFP- (**B**), Cre-NLS-HiBiT, Jun-HiBiT, Myc-HiBiT, MYL3-HiBiT, NANOG-HiBiT, YAP8SA-HiBiT, or mock transfected (**C**); raw luminescence data were normalized to time 0, dots representing the mean of three biological replicates per time point. (**D**) Fold change in AUC of curves from 0 to 4 h or to 18 h of three biological replicates was obtained by normalizing to reference sample MYL3 and compared by two-way ANOVA with matched values. Cellular HiBiT lytic assay data for hESC-CMs (**E**) and A549 (**F**) transfected with Cre-NLS-HiBiT, eGFP-HiBiT, Jun-HiBiT, Myc-HiBiT, MYL3-HiBiT, NANOG-HiBiT, YAP8SA-HiBiT, or mock transfected and lysed 4 h post-transfection. Luminescence signal of three biological replicates was normalized to that of reference sample MYL3 and statistical analysis was performed by one-way ANOVA with multiple comparisons. *P< 0.05, **P< 0.005, 0.0001 < ***P< 0.0005, and ****P< 0.0001.

Given that eGFP is highly engineered and its translation is extremely efficient, six additional mRNAs encoding HiBiT-tagged non-engineered proteins, were generated to investigate translation dynamics. Specifically, we chose a non-human protein, Cre recombinase (fused to an NLS), and five human genes: transcription factors c-JUN, c-MYC (hereon Jun and Myc), and NANOG; constitutively active transcriptional regulator YAP8SA; and structural protein Myosin light chain 3 (MYL3). All protein fusion contained the HiBiT tag at the C-terminus, and mRNA constructs retained the identical Cap (ARCA), 5′UTR, linker and HiBiT sequence and 3′UTR, as described for eGFP-HiBiT. To demonstrate that the correct proteins were encoded, mRNAs were translated for 120 min using the RRL system and resulting protein samples were processed for western blotting. Incubation of the membrane with LgBiT and Furimazine confirmed that mRNA translation resulted in proteins of the expected size for all constructs (Fig. 3A). In HEK293 LgBiT cells, eGFP-HiBiT encoding mRNA resulted in a rapidly translated protein that quickly reached a plateau when protein synthesis and degradation reached a steady state (Fig. 3B). The dynamic of eGFP-HiBiT mRNA translation in cells significantly outperformed that of any other gene (Supplementary Fig. 2A and B), with an increase in fold change in AUC over the next best expressed gene, MYL3-HiBiT, of 2.8-fold over 4 h, and 4.6-fold over 18 h (Supplementary Fig. 2C). Excluding eGFP-HiBiT from our analysis and comparing the dynamics of mRNA translation and protein turnover, MYL3-HiBiT resulted in a rapid increase in luminescent signal that appeared to plateau after 6 h, which was significantly higher than all remaining genes as measured by fold change in RLU (Supplementary Fig. 2D and E), and in AUC (*P*< 0.0001, Fig. 3D). Cre-NLS-HiBiT expression resulted in comparable levels of expression to Jun-HiBiT, NANOG-HiBiT, and YAP8SA-HiBiT (Fig. 3C and D). Interestingly, all three genes demonstrated a similar dynamics curve, which peaked between 4 and 6 h and dipped over time (Fig. 3C); however, the peak intensity reached varied between constructs. Jun-HiBiT reached the highest luminescence signal of the three transcription factors and significantly higher than Myc-HiBiT over 4 and 18 h (*P*< 0.0001, Fig. 3D) and NANOG-HiBiT over 18 h (*P*= 0.0395). NANOG-HiBiT also demonstrated significantly higher expression than Myc-HiBiT over 4 h (*P*= 0.0119, Fig. 3D). Myc-HiBiT levels were the lowest of all genes tested at 4 and 18 h (Fig. 3C and D). Finally, YAP8SA demonstrated a slower initial increase in signal than Cre-NLS-HiBiT, Jun-HiBiT, and NANOG-HiBiT, but uniquely its signal was seen to continue increasing over time (Fig. 3D). To assess the contribution of mRNA levels in cells to the signal output, we collected HEK LgBiT cells at 4 and 18 h post-transfection, isolated total cellular mRNA, and performed qRT-PCR analysis. Results reveal no significant difference in mRNA levels of eGFP-HiBiT, Jun-HiBiT, Myc-HiBiT, NANOG-HiBiT, YAP8SA-HiBiT, and Cre-NLS-HiBiT at 4 or 18 h, in spite of the huge differences in luminescence output and dynamics described (Supplementary Fig. 2F). MYL3-HiBiT mRNA levels were significantly higher than Jun-HiBiT, Myc-HiBiT, NANOG-HiBiT, and YAP8SA-HiBiT at 4 h, but this difference was abolished at 18 h.

To establish whether the results obtained in HEK LgBiT cells were unique to the cell line or descriptive of translational dynamics of these gene regardless of cellular context, we transfected the same mRNA constructs in same quantities in hESC-CMs and A549 cells. Since these cell lines do not constitutively express LgBiT, we performed the cellular lytic version of the assay, where cells were lysed and LgBiT and Furimazine substrate were added to the lysates to detect the luminescent signals produced by translated HiBiT mRNA. Due to the lack of temporal information in this assay, based on the HEK LgBiT data, we chose to conduct the experiment for the length of time that resulted in all constructs being detected at peak level, therefore cell lines were lysed and luminescent signal was measured at 4 h post-transfection. In hESC-CMs (Fig. 3E) and A549 (Fig. 3F), all genes resulted in significantly higher signal than mock, demonstrating the specificity of the assay. eGFP-HiBiT out-performed any other HiBiT-encoding mRNA, with a 2.6- and 4.1-fold increase over MYL3 in hESC-CM and A549 respectively, confirming the data from HEK LgBiT cells. Additionally, similar to HEK LgBiT cells, the trend of relative expression of Cre-NLS-HiBiT, Jun-HiBiT, Myc-HiBiT, and NANOG-HiBiT was confirmed, with Jun-HiBiT demonstrating highest signal at 4 h, followed by Cre-NLS-HiBiT and NANOG-HiBiT with comparable levels and Myc-HiBiT being the lowest. In both additional cell lines YAP8SA-HiBiT levels were higher than those of of Cre-NLS-HiBiT, Jun-HiBiT, Myc-HiBiT, and NANOG-HiBiT, which is in contrast to the HEK LgBiT data. Overall, the data demonstrates that the HiBiT assay can detect gene-specific differences in translational and post-translational regulation and highlights how no gene tested results in comparable dynamics of expression to eGFP.

**Figure 4.**
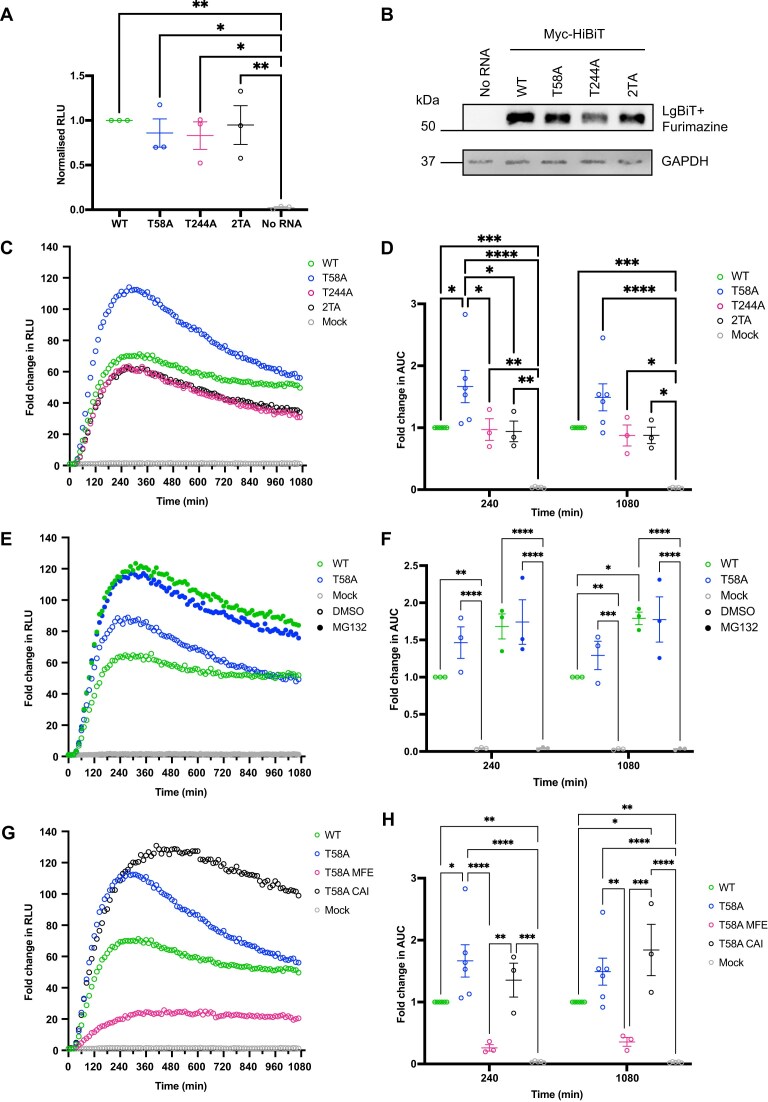
HiBiT-encoding mRNA reports on optimization of Myc coding sequence. (**A**) mRNA translation of Myc with threonine (T) to alanine (A) substitutions at key phosphodegron sites was compared in the RRL system by incubating the HiBiT mRNA for 120 min. Scatter plot depicting RLU of three biological replicates normalized to WT Myc-HiBiT, mean, and SEM. (**B**) Western blot of pooled samples from the three replicates, displaying GAPDH and Myc-HiBiT detected by LgBiT and Furimazine. (**C**) Live cellular kinetic assay in HEK293 LgBiT cells transfected with Myc-HiBiT or Mock, data normalized to t0; dots representing the mean of at least three biological replicates per time point. (**D**) AUC were calculated for data over 4 and 18 h and normalized to WT to depict changes in Myc-HiBiT signal and compared using two-way ANOVA with matched values. (**E**) HEK293 LgBiT transfected with Myc-HiBiT WT or T58A were co-treated with DMSO (0.005%, empty circles) or MG132 (Cf = 4 μM, full circles) at seeding, and expression was measured through time and normalized to t0 and compared to WT by one-way ANOVA. (**F**) AUC was calculated for data at 4 and 18 h and was normalized to WT DMSO and compared by two-way ANOVA with matched values. (**G**) Two codon optimization strategies applied to Myc T58A and translation were compared in the live cellular kinetic assay in HEK293 LgBiT cells. Fold change in RLU from time 0 is shown with dots representing the mean of three or six biological replicates per time point. (**H**) AUC data over 4 and 18 h normalized to WT depicts changes in Myc-HiBiT levels and compared using two-way ANOVA with matched values. *P< 0.05, **P< 0.005, 0.0001 < ***P< 0.0005, ****P< 0.0001. WT = wild-type Myc (green); T58A = threonine-to-alanine substitution at T58 of Myc (blue), T244A = threonine-to-alanine substitution at T244 (magenta in C,D); 2TA = threonine-to-alanine substitution at T58 and T244 (black in C and D); T58A MFE = minimum-free-energy optimization of T58A Myc (magenta in G and H), T58A CAI = codon-adaptation-index optimization of T58A Myc (black in G and H).

We next investigated the HiBiT assay’s sensitivity to subtle substitutions in coding sequences. We focused on the Myc coding sequence since it was the lowest expressing of all mRNAs (Fig. 3C) and optimized it for protein stability and translation efficiency. The rapid protein turnover of Myc is, in part, mediated by the phosphodegron site threonine (T) 58, which is a recognition site for E3 ligase, FBW7 [37–39]. More recently a second phosphodegron for regulation of Myc by FBW7 has been discovered [40] at threonine 244. To determine the effects of phosphoablation on these phosphodegron sites of Myc-HiBiT, we generated three Myc mutants—T58A, T244A, and 2TA (T58A and T244A). Using the RRL system, no significant difference was recorded when comparing mutants to WT Myc (Fig. 4A and B), suggesting all mRNAs are equally translated. Myc-HiBiT mRNAs were then tested in the live kinetic cellular assay. All versions of Myc were rapidly translated with peak signal reached around 4 h (Fig. 4C). However, the signal rapidly declined suggesting that the longevity of the protein signal may depend on the protein half-life of the encoded POI. Interestingly, none of the stabilizing mutations of Myc-HiBiT resulted in an improved, more sustained signal at 18 h but were all significantly higher than mock (Fig. 4D, and Supplementary Fig. 3A and B). When comparing the AUC of mutants to that of WT at these time points (Fig. 4D), T58A resulted in a significantly higher (*P*= 0.0129) average of 67% increase in stability over 4 h, which is reduced to 49% when assessing the dynamics over 18 h. Myc T244A only resulted in a 3% reduction in AUC over 4 h and 13% over 18 h. The double phosphodegron mutant 2TA had comparable values to T244A over 4 h (average 6% less than WT) and 18 h (average 12% less than WT).

To prove that the observed difference between Myc WT and T58A is due to the changes in post-translational regulation, HEK293 LgBiT cells were treated with a sublethal dose of proteasome inhibitor MG132 (4 μM) or vehicle control DMSO at the time of transfection (Fig. 4E, and Supplementary Fig. 3C and D). Proteasomal inhibition was demonstrated by the resulting increased WT Myc-HiBiT signal, with AUC elevated by 68% over 4 h and 79% over 18 h (*P*= 0.0185 and Fig. 4F), due to accumulation from lack of degradation. As predicted, at 4 h, there is an overlap in WT and T58A signal upon MG132 treatment, indicating that the higher peak of T58A in the DMSO control is due to reduced degradation of the mutant compared to the WT at comparable exponential translation rates.

Since regulatory elements have been reported to control Myc mRNA turnover [39, 41–44], Myc-HiBiT mRNA optimization strategies were shifted to improving mRNA translation and stability. Two mRNA optimization strategies, aimed at improving mRNA stability and codon optimality [9], were applied to the best stability mutant, Myc T58A-HiBiT. Applying the published algorithm, we generated mRNA encoding for the sequence with the highest CAI and the highest MFE. Specifically, the MFE and CAI indexes respectively were: −509.00 kcal/mol; 1.000 for T58A CAI; −944.40 kcal/mol; 0.737 for T58A MFE. Importantly, only the open reading frame of Myc T58A sequence was optimized (−457.60 kcal/mol; 0.819 MFE and CAI indexes), whilst the UTRs, poly(A), linker and HiBiT sequence remained unchanged to allow for accurate comparison to WT and T58A Myc-HiBiT mRNA. Optimization of the mRNA stability with T58A MFE impeded efficient translation (Fig. 4G, and Supplementary Fig. 3E and F), reducing the AUC of Myc-HiBiT by 74% compared to WT over 4 h, and 65% over 18 h, and significantly lower than the non-optimized T58A (Fig. 4H, *P*< 0.0001 and *P*= 0.0011 at each time point, respectively). However, despite the reduced protein abundance, over the time course of the experiment, there was no rapid decline in signal suggesting an enhanced mRNA stability. Improving the codon adaptability index (T58A CAI) resulted in a delayed peak at 8 h compared to WT and T58A Myc-HiBiT and a more sustained signal over time (Fig. 4G). Specifically, over 4 h, T58A CAI showed an average 35% increase in AUC over WT compared to the 67% of T58A, but over 18 h there is a statistically significant (*P*= 0.0228) 84% increase in AUC over WT with T58A CAI against the 49% of T58A.

### Modified nucleotides

**Figure 5.**
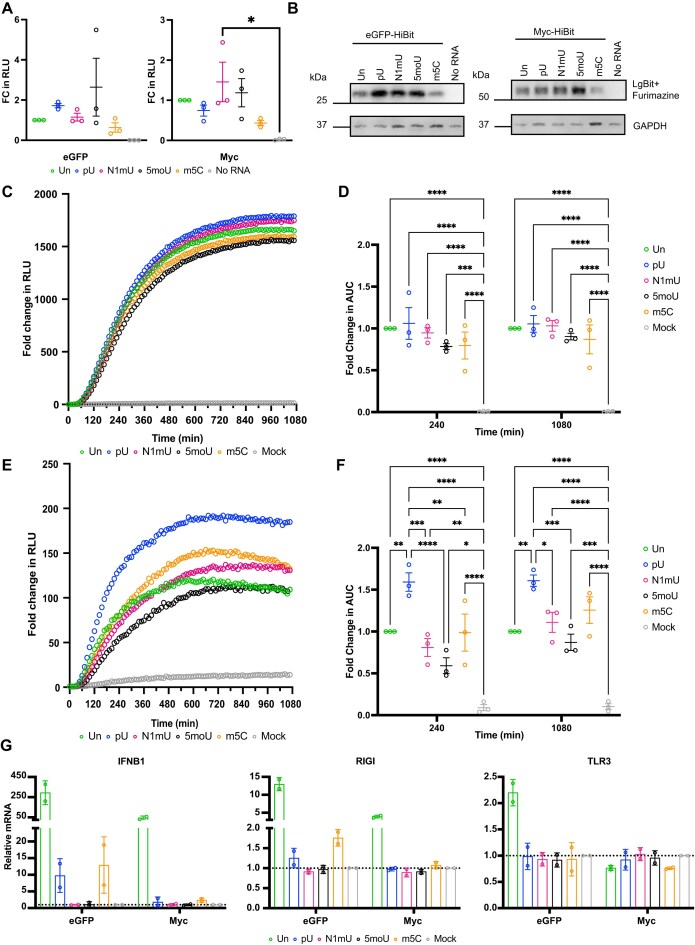
Effect of nucleotide modifications on HiBiT-encoding mRNA translation depends on the encoded POI. (**A**) mRNA translation of eGFP-HiBiT or Myc-HiBiT mRNA containing unmodified or 100% substitution with modified nucleotides was compared in the RRL system by incubating the HiBiT mRNA for 120 min. Scatter plot depicting RLU of three biological replicates normalized to unmodified (Un) eGFP-HiBIt or Myc-HiBiT, mean, and SEM. (**B**) Western blot of pooled samples from three RRL replicates showing GAPDH and eGFP-HiBiT or Myc-HiBiT detected by LgBiT and Furimazine. (**C**) Live cellular kinetic assay in HEK293 LgBiT cells transfected with eGFP-HiBiT (unmodified and modified) or mock, data normalized to t0; dots representing the mean of three biological replicates per time point. (**D**) Fold change in AUC at 4 and 18 h was normalized to unmodified to depict changes in eGFP-HiBiT signal and compared by two-way ANOVA with matched values. (**E**) HEK293 LgBiT transfected with Myc-HiBiT (unmodified or modified) or mock and fold change in RLU from t0 was plotted (**F**) Fold change in AUC at 4 and 18 h was obtained by normalization to unmodified Myc-HiBiT and compared using two-way ANOVA with matched values. (**G**) Induction of immunogenic gene expression (IFNB1, RIGI, and TLR3) in HEK293 LgBiT cells transfected with unmodified or modified eGFP-HiBiT or Myc-HiBiT was assessed by qRT-PCR and normalized against mock sample for two biological replicates. *P< 0.05; **P< 0.005; 0.0001 < ***P< 0.0005; ****P< 0.0001. Un = unmodified (green); pU = pseudouridine-5′-triphosphate (blue); N1mU = N1-methylpseudouridine-5′-triphosphate (magenta); 5moU = 5-methoxyuridine-5′-triphosphate (black); m5c = 5-methylcytidine-5′-triphosphate (orange).

The impact of nucleoside modifications on the expression of the encoded protein is likely dependent on the gene of interest. Employing the HiBiT system, full substitutions of unmodified uridine or cytidine with modified analogues were implemented during the *in vitro* transcription reaction of the highest and lowest expressing mRNAs eGFP-HiBiT and Myc-HiBiT (Fig. 3B and C). Specifically, the contribution of pseudouridine-5′-triphosphate (pU), N1-methylpseudouridine-5′-triphosphate (N1mU), 5-methoxyuridine-5′-triphosphate (5moU), and 5-methylcytidine-5′-triphosphate (m5C) on translation were investigated in the HiBiT assay system. Unmodified (Un) or modified RNAs (modRNA) were translated *in vitro* with the RRL system for 120 min, and resulting HiBiT-tagged proteins were detected via a luminometer (Fig. 5A) and by western blotting (Fig. 5B). All modifications to eGFP-HiBiT and Myc-HiBiT resulted in a translated protein. For both mRNAs, modification with m5C resulted in a non-significant reduction in translated protein, with 37% and 57% respective average decrease in luminescent signal (Fig. 5A). This trend was confirmed by western blotting (Fig. 5B).

Next, we assessed the effect of the modifications on mRNA translation dynamics *in cellulo*. Incorporation of modifications into eGFP-HiBiT did not affect its translational dynamics profile, which displayed rapid accumulation and plateauing. All modifications showed rapid accumulation of eGFP-HiBiT signal (Fig. 5C) with no significant changes compared with unmodified mRNA (Fig. 5D; Supplementary Fig. 4A and B). When comparing the effects of nucleotides on Myc-HiBiT mRNA expression, a greater distinction in the effect of the modifications was observed (Fig. 5E and F; Supplementary Fig. 4C and D). Myc-HiBiT expression was recorded for all mRNAs regardless of modification status, with pU substitution producing both a faster initial burst of Myc-HiBiT synthesis and a more prolonged steady-state of synthesis over the time course of the assay (Fig. 5E), resulting in an increased average fold-change in AUC of 60% over 4 h (*P*= 0.0078), and 61% over 18 h (*P*= 0.0060) when compared to unmodified Myc-HiBiT (Fig. 5F). pU substitution resulted in significant improved translation dynamics compared to all modifications over 4 h and over all other uridine substitutions throughout 18 h (Fig. 5F).

Since improved translation rates do not solely dictate the choice of modification, we verified the immunogenic response to all mRNAs by qRT-PCR. We selected three genes that encode for cytosolic RNA sensors (retinoic acid-inducible gene I, RIGI; TLR3) and downstream effector (interferon beta 1, IFNB1). mRNAs encoded with unmodified nucleotides resulted in a large immunogenic response, which was substantially higher for eGFP-HiBiT than Myc-HiBiT modRNA (Fig. 5G). The inclusion of modified nucleotides blunted the immunogenic response to varying degrees depending on modification (Fig. 5G). Based on these results, the impact of the choice of cap on immunogenic response to unmodified eGFP-HiBiT was assessed by qRT-PCR and incorporating additional RNA sensors and effector genes: interferon induced with helicase C domain 1 (IFIH1), C-C motif chemokine ligand 5 (CCL5), and tumour necrosis factor (TNF). Comparison of immune response induced by mRNA with ARCA and CleanCap demonstrated a significant reduction in immunogenic response with CleanCap, which was not significantly different to mock infected HEK293 LgBiT cells (Supplementary Fig. 4E).

### Poly(A) length

**Figure 6.**
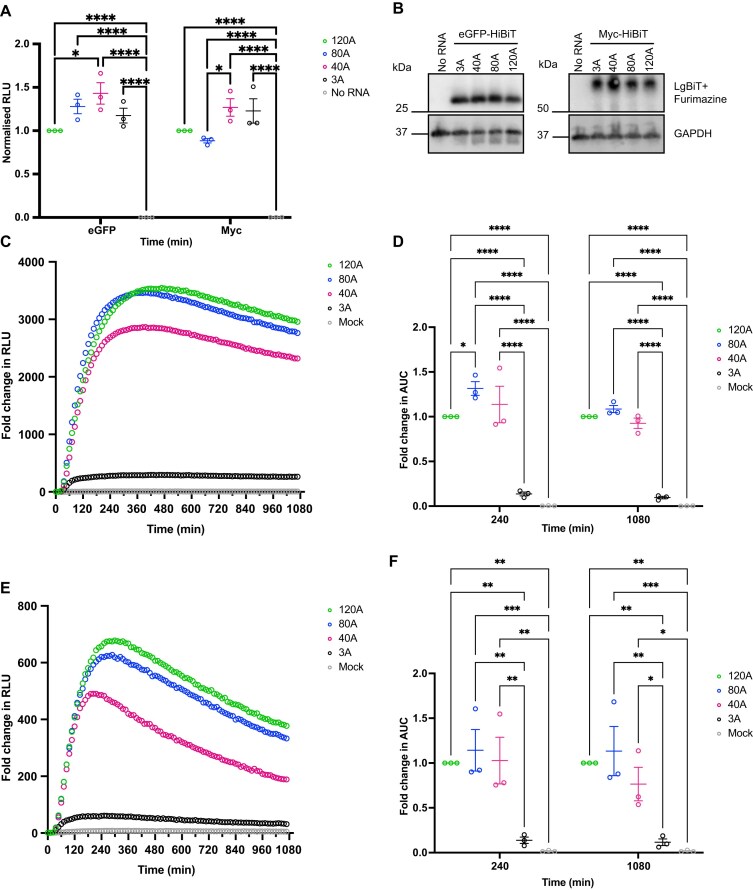
Effect of poly(A) length on HiBiT-encoding mRNA translation is independent of the encoded POI. (**A**) mRNA encoding eGFP-HiBiT or Myc-HiBiT with poly(A) tails of 3 (black), 40 (magenta), 80 (blue), and 120 (green) adenosines was compared in the RRL system by incubating the HiBiT mRNA for 120 min. Scatter plot depicting RLU of three biological replicates normalized to 120A eGFP-HiBiT or Myc-HiBiT, mean and SEM. (**B**) Western blot of pooled RRL samples with GAPDH as a loading control and eGFP-HiBiT protein detected by LgBiT and Furimazine. (**C**) Live cellular kinetic assay in HEK293 LgBiT cells transfected with eGFP-HiBiT with differing poly(A) lengths or Mock. RLU per sample was normalized to time 0 to produce fold change in RLU; dots representing the mean of three biological replicates per time point. (**D**) AUC of data over 4 and 18 h was normalized to eGFP-HiBiT 120A to measure fold change in the HiBiT signal and compared using two-way ANOVA with matched values. (**E**) Live cellular kinetic assay in HEK293 LgBiT cells transfected with Myc-HiBiT with differing poly(A) lengths or mock. Fold change in RLU from time 0 is depicted, with dots representing the mean of three biological replicates per time point. (**F**) Fold change in AUC over 4 and 18 h was calculated by normalizing to 120A Myc-HiBiT and compared using two-way ANOVA with matched values. *P< 0.05; **P< 0.005; 0.0001 < ***P< 0.0005; ****P< 0.0001.

To assess and measure the effect of poly(A) tail length on POI mRNA translation, four different mRNAs were generated encoding eGFP-HiBiT and Myc-HiBiT with poly(A) lengths of different sizes: 3, 40, 80, or 120 adenosines. All mRNAs were translated in parallel for a total of 120 min in the RRL system. The resulting luminescence readings were normalized to the average of the 120(A) sample per replicate (Fig. 6A). Proteins were also detected by western blotting (Fig. 6B). All Poly(A) lengths tested achieved mRNA translation in the RRL system, an improvement of 40(A) over 120(A) for eGFP (*P*= 0.006) and 40(A) and 3(A) over 80(A) for Myc (*P*= 0.0167, *P*= 0.0365, respectively) was observed.

Live kinetic recordings in HEK293 LgBiT cells confirmed that the rapid translation of eGFP-HiBiT post-transfection is common to all poly(A) forms except where the poly(A) tail is reduced to 3 adenosines (Fig. 6C and D). In cells, 80A poly(A) demonstrated a statistically higher AUC than 120A over 4 h (31%, *P*= 0.0438); however, this is lost when comparing the AUC for the full kinetic profile over 18 h (Fig. 6D) because the 80A tailed-mRNA reaches peak signal faster than the 120A mRNA, which maintains higher signal for longer (Fig. 6C). The short 3A poly(A) almost ablated the translation of eGFP-HiBiT (Fig. 6C; Supplementary Fig. 5A, B), the AUC over 4 and 18 h being significantly lower than that of any mRNA and 86% lower than 120A over 4 h and 90% over 18 h (*P*< 0.0001) and not significantly higher than that of the mock-transfected control (Fig. 6D).

The translation dynamics of mRNA encoding for Myc-HiBiT recapitulated the observed impact of varying poly(A) lengths (Fig. 6E and F; Supplementary Fig. 5C and D), where a poly(A) of 3 adenosines ablated Myc-HiBiT expression to 86% over 4 h (*P*= 0.0059) and 88% over 18 h compared to 120 adenosine tail (*P*= 0.0047, Fig. 6E).

To assess whether changes in HiBiT signal detected is due to differences in HiBiT-encoding mRNA in cells, qRT-PCR was performed by collecting HEK293 LgBiT cells transfected with eGFP-HiBiT with differing poly(A) lengths at 4 and 18 h post-transfection. qRT-PCR data showed no difference in levels of eGFP-HiBiT mRNA with 40, 80, or 120 adenosine-long tail at either timepoint but these were statistically higher than mock (Supplementary data Fig. 5E). Interestingly, in the case of eGFP-HiBiT mRNA with a 3A tail, the mRNA either did not transfect into cells or was degraded rapidly, accounting for the limited expression in the live cell assays.

## Discussion

The development of mRNA-based therapeutics has increased exponentially in recent years, and it has become clear that a crucial step in the development pipeline is mRNA sequence design and optimization. A large effort has been devoted to the generation of computational tools to predict the optimal structure and composition of the RNA, with a particular focus on codon optimization [9, 8], and the experimental validation has largely relied on existing assays, which often employ reporter proteins (GFP and luciferase) or the SARS-CoV-2 spike protein [9, 8, 7]. In this work, we implement the HiBiT tag in a pipeline for studying the dynamics of translation of exogenous mRNA *in vitro*, in the RRL system, and *in cellulo*, in HEK293 cells constitutively expressing LgBiT. Importantly, the addition of the small component of the luciferase as a tag did not affect *in vitro* translational dynamics and offered a robust luminescent read-out specific to the tagged POI (Fig. 1). Additionally, a luminescence but not a fluorescence read-out is compatible with the RRLs with a signal:noise that results in an assay with a *Z*′ = 0.8. The robust assay is compatible with applications in multi-well formats and, therefore faster and higher throughput than canonical assays for protein detection, which rely on antibody-mediated or radioactive labelling detection methods. The addition of the tag also permits the detection of the mRNA-driven expression of the POI over time in live cells constitutively expressing LgBiT, treated with the luciferase substrate (Figs. 2–6). The live luminescence recordings are vital in determining the expression window of the therapeutic mRNA, without the need to extrapolate information from reporter genes like engineered fluorophores or luciferases that translate very efficiently and are often stable proteins. In agreement, our data shows that the dynamics of expression between six POIs, Cre-NLS-HiBiT, eGFP-HiBiT, Jun-HiBiT, Myc-HiBiT, MYL3-HiBiT, NANOG-HiBiT, and YAP8SA-HiBiT are different (Fig. 3B–D, Supplementary Fig. 2). If no LgBiT-expressing cells are available, assessment of POI-HiBiT in cells can be carried out using the Nano-Glo HiBiT lytic detection system (Promega), where cells are lysed and LgBiT and the substrate are added to the lysates. The lytic method for detection of mRNA-encoded POI-HiBiT has been recently employed for validation of an open-source computational mRNA optimization tool [8], specifically for dose-response studies of mRNA administration at a set endpoint. Here, we demonstrated its use in hESC-CMs and A549 (Fig. 3E and F, respectively). The strategy is high-throughput and adaptable to different cell types but provides no information on the dynamics of expression of the administered mRNA. Indeed, we had to extrapolate the time point based on the time-resolved data obtained in HEK293 LgBiT cells, confirming the trends in expression between the six genes at a set time point of 4 h. While time-course experiments can be carried out with the lytic system, it requires sampling of replicates at different time points instead of live monitoring of one individual replicate over the time course of the assay, increasing the likelihood of variability between experimental replicates. Given the availability of techniques to generate stable cell lines, the engineering of LgBiT knock-in cells in the model of interest unlocks the ability to conduct mRNA kinetic studies specific to the cell of interest and compare such dynamics between differing cell types.

To demonstrate the sensitivity of the mRNA-HiBiT assay pipeline, we modified coding and non-coding elements of the mRNA and verified its ability to detect changes in mRNA translation. We confirmed the ability of both RRL and live cell assays to measure translation by comparing capped and uncapped mRNA, which only resulted in luminescence being recorded in the presence of the cap (Fig. 2A–C, G, and H). We next compared cap1 (CleanCap) to cap0 (ARCA) (Fig. 2A–C). Significant translational benefit from cap1 has been seen in cells such as the murine immature dendritic cell line, JAWS II, and macrophages [45–47]. We detected little difference in translation dynamics when comparing ARCA and Clean Cap in HEK293s (Fig. 2G–H), suggesting changes in translation dynamics may be cell-type dependent [45, 46] and target cells should be evaluated. Cap1 has been shown to reduce immunogenic response [48, 49] because 2′-*O*-methylation of the first nucleotide can help distinguish self RNA from non-self RNA by preventing recognition by RIGI and IFIT1 (interferon‐induced protein with tetratricopeptide repeats 1). This was confirmed by our qRT-PCR data where we compared the induction of expression of immunogenic genes in HEK293 LgBiT cells transfected with ARCA or CleanCap incorporating eGFP-HiBiT mRNA or mock (Supplementary Fig. 4E). Therefore, cap1 possesses an additional benefit of reducing immune silencing over the cap0 structure which is particularly attractive for *in vivo* use.

Engineering a hairpin structure within the 5′ of the mRNA significantly impacted the POI translation in cells, to the same extent of removal of the 5′UTR. Previous work suggests that mRNAs with limited thermodynamically stable secondary structures (<-30 kcal/mol MFE) allow for efficient translation, whereas more stable secondary structures (>-50 kcal/mol) stall the 40S ribosome and decrease translation efficiency [50]. In agreement, an mRNA with a 5′UTR containing limited thermodynamically stable secondary structures (∼–10 kcal/mol) was efficiently translated whereas translation was significantly attenuated with an mRNA containing a 5′UTR-hairpin with an MFE close to −50 kcal/mol. These data demonstrate that this assay can detect changes in translation resulting from different ranges of mRNA stability. Interestingly, the reduced translation was not observed in RRLs. Considering the nuclease-treated RRL has been depleted of all endogenous mRNAs, the assay does not recapitulate competition between different mRNAs for the ribosome and cap-poly(A) synergy [19, 20], which may account for the differences observed. These data highlight the need for in-cell assays that allow discrimination of possible blocks to translation.

A key advantage of our live kinetic assay is its ability to measure both translation rates, in the early phases of the experiment, and protein turnover, after the initial burst of translation. To demonstrate this, we compare the translational dynamics of eGFP-HiBiT and five additional coding sequences encoding for different genes. Cre-NLS, a bacterial enzyme capable of recognizing and excising DNA at loxP sites was chosen as non-optimized protein that is not expressed in human cells. Human structural protein MYL3, not expressed in HEK293 cells but in cardiomyocytes, was chosen due to its very long reported half-life (over 10 days [51]). Highly regulated human transcription factors Jun, Myc, and NANOG, all capable of inducing genome-wide transcriptional changes and with reported shorter half-lives (Myc 20–40 min [52, 53], Jun 90–120 min [54, 55], NANOG 120 min in hESCs [56]). It is worth noting that protein turnover rates can vary greatly between cell types, and in HEK293 LgBiT cells with CRISPR HiBiT knock-in at the C-terminal end of endogenous Myc, we estimated a half-life of 195 min (Supplementary Fig. 6). Finally, we included the human transcriptional regulator YAP. Since the half-life is linked to its phosphorylation state and cell density, with WT YAP reported to have a half-life of ∼2 h and constitutively active phosphoablation mutant 5SA still detected after 8 h of cycloheximide chase [57], we chose the constitutively active 8SA, which should have a comparable, if not improved, half-life as the 5SA mutant.

Confirming our previous observation [33] the expression of eGFP-HiBiT in cells is far greater than any other gene (Fig. 3B and Supplementary Fig. 2A–C), and the signal is prolonged. MYL3-HiBiT displays similar translational dynamics to eGFP-HiBiT, although with a nearly five-fold greater change in AUC of the latter compared to the former (Fig. 3B and C; Supplementary Fig. 2C). Signal from Cre-NLS-HiBiT was significantly lower to that of MYL3 and eGFP at comparable levels of mRNA (Supplementary Fig. 2F), likely because the sequence used is not that of codon-optimized iCre [58] and differences in protein half-life. The dynamics of expression of the HiBiT-tagged transcription factors Jun, Myc, and NANOG depicted peaks at 4 h that decline rapidly (Fig. 3C), with the difference in maximal luminescence reached varying between the transcription factors. Since comparable levels of mRNA of these genes were detected by qRT-PCR (Supplementary Fig. 2F), the difference in translational dynamics is likely due to the differences in protein turnover. Indeed, Jun and NANOG, that have comparable reported half-lives of around 2 h, did not display significantly different AUCs, instead both AUCs were significantly higher over 4 h than that of Myc, that has a reported half-life of less than an hour. Finally, while YAP8SA-HiBiT mRNA did not result in rapid accumulation of the signal, this is the longest mRNA tested and the biggest resulting protein at nearly 75 kDa. Interestingly, the levels of YAP8SA-HiBiT signal appeared to continue increasing for the 18 h of the experiment (Fig. 3C), and with its mRNA level being comparable at 4 and 18 h, this is likely due to the long protein half-life.

An advantage of using the HiBiT system is that it enables the detection of the exogenously expressed POI, which can be difficult to detect if the endogenous equivalent is expressed in the cell line of interest. Indeed, no HiBiT signal is recorded in mock transfection in favour of, for example, Myc-HiBiT transfected HEK293 LgBiT cells that do express abundant levels of endogenous Myc (Fig. 4).

As previously mentioned, the half-life of Myc is estimated to be around 20–40 min [52, 53]. Mutation of T58 to a non-phosphorylatable alanine (T58A) leads to an over three-fold increase in Myc half-life [52], which promotes growth and proliferation [59] and is a mutational hotspot in lymphomas [60, 61]. A second phosphodegron, T244, has also been identified, but less is known regarding its ability to stabilize Myc protein [40]. In our HiBiT assays, all phosphoablation mutants translated equally in the RRL system (Fig. 4A and B). In HEK293 LgBiT, only Myc-T58A-HiBiT, displayed increased expression over the WT Myc-HiBiT (Fig. 4C). Interestingly, this was true at the peak of expression when T58A resulted in a 67% increase in Myc-HiBiT signal over 4 h, which was not sustained over time. Within the first 4 h, the translation rate is in the exponential phase and likely greater than the degradation rate, therefore the effect of changes in protein stability can be observed, as confirmed by proteasomal inhibition (Fig. 4E and F). Importantly, if these studies had been conducted as a single end-point evaluation at 18 h, all versions of Myc would have been deemed comparable, despite the known phenotypic differences that Myc-T58A triggers [59–61]. Since other regulatory elements that dictate Myc turnover exist [60, 62, 63], further sequence modifications were employed to determine whether a more stable translational output was achievable. Applying a published, open-source algorithm [9] to the best-performing stability mutant (T58A), codon optimization and mRNA stability strategies were implemented and tested using the HiBiT assay system. In HEK293 cells, translation of Myc T58A-HiBiT was greater for a CAI-optimized sequence, producing a delayed peak and prolonged translation with an 84% increase in signal throughout the 18 h post-transfection compared to non-optimized WT and 49% to T58A Myc-HiBiT (Fig. 4G and H). Contrarily, MFE optimization led to reduced translation with a 65% reduction of Myc-HiBiT signal. However, MFE optimization did lead to a more stable, albeit lower, Myc-HiBiT signal, within the timeframe of the described experiment. These observations agree with previous reports that highly structured RNA sequences can enhance mRNA stability in cells, but the highly structured RNA decreases the ability of the cellular translation machinery to process the RNA [7]. Interestingly, both optimization strategies combined were insufficient to bring Myc close to the expression levels and steady state of protein synthesis and degradation that can be sustained over time, as seen with eGFP. Together these findings highlight how the optimization strategy should be tailored to the encoded POI and its biological output. Overall, our data supports the ability of this HiBiT assay system to report on changes to the coding sequence, both with regards to improving mRNA translatability through codon optimization strategies and protein engineering through stability mutants.

An essential aspect of the development of mRNA-based therapeutics is the incorporation of modified nucleotides, which are chosen based on their effect on the translation efficiency of the mRNA and their ability to reduce immunogenic responses [5, 64, 65]. Importantly, our data demonstrate that the choice of modification will depend on the encoded gene and therefore modified nucleotides should be tested on the therapeutic mRNA, not a reporter protein. By comparing modified nucleotides incorporated in mRNA encoding eGFP-HiBiT and Myc-HiBiT, we found the choice of modifications only minimally affected eGFP-HiBiT expression but greatly affected Myc-HiBiT translation, with incorporation of pU resulting in a higher and more sustained expression over time (Fig. 5), in line with previous literature. It is important to note that the data presented in this work does not address how the different modifications may affect translation rates in alternate cell types. Stable cell lines engineered to express LgBiT would be valuable to enable comparisons to be made via the HiBiT system. One aspect of the effects of nucleotide modification that we did not address here, is translational fidelity. Recently it was shown that N1mU causes + 1 ribosomal frameshifting but only in the presence of ribosome slippery sequences and ribosomal stalling [66]. Importantly, while N1mU modifications may affect the translation fidelity of specific sequences overall, they have been shown to maintain tRNA selection and produce faithful proteins [67], highlighting the varied impact of modified nucleotides on mRNA fidelity and thus the need for specific sequence optimization in different contexts with the end goal in mind.

Finally, we assessed the contribution of differing poly(A) lengths to mRNA translation *in vitro* and *in cellulo*. In the RRL system, no advantage of increased Poly(A) length was measured, when ARCA cap is present (Fig. 5A and B), but loss of cap could not be rescued by the addition of a long (120 adenosine) poly(A) tail (Fig. 2A–C). Therefore, for protein synthesis applications, a tail with as little as three adenosines is sufficient for efficient translation of capped mRNA. This is in agreement with previous reports which demonstrated that in nuclease-treated RRLs, such as the Flexi Rabbit Reticulocyte Lysate Kit, poly(A) tail length had minimal effects on the translation of capped mRNA [68, 69]. While we cannot confirm the length of the poly(A) of our constructs once in cells, we showed that similar levels of mRNA were detected at 4 and 18 h with 40, 80, and 120 adenosine-long tails (Supplementary Fig. 5E). In cells, minimal translation was recorded from constructs containing mRNA with a 3-adenosine poly(A), likely due to fast degradation of these mRNA once inside the cells, as denoted by qRT-PCR where significantly lower levels of 3A-tailed mRNA were recorded compared to 40A, 80A, or 120A (Supplementary Fig. 5E). Per the literature, high-affinity binding of PABPC to poly(A) tails requires ∼12 nucleotides, with a bound PABPC covering around 30 adenosines in length [70, 71], meaning an mRNA with 40A tail will only be bound by one PABC protein versus two or more which have been shown to increase translation efficiency [72–74]. mRNA encoding eGFP-HiBiT or Myc-HiBiT with a poly(A) tail of at least 40A was efficiently translated likely due to the binding of a PABPC molecule. No difference was detected between the translation of mRNAs with a tail permissive of binding of two (80A) or more (120A) PABCP, indicating that for mRNA therapeutic design, 80A tails are sufficient. These data demonstrate the ability of the HiBiT assay pipeline to detect translation rate differences brought about by differing poly(A) lengths, recapitulating published data.

Overall, our findings demonstrate that the high-throughput HiBiT pipeline that we describe facilitates mRNA translation measurement both *in vitro* and *in cellulo*. While the *in vitro* translation system of the RRL yields detectable translation changes by coding and non-coding elements of an exogenous mRNA, it is not a reliable predictor of efficient mRNA translation in live cells, possibly due to discrepancies between model systems, but can prove useful as a first-line screening tool. Implementing a live cell time course assay, as we describe, overcomes this limitation and unlocks essential information on the dynamics of protein synthesis and degradation of a POI encoded by an exogenous mRNA. Importantly, the live kinetic assay can record differing windows of peak expression of a therapeutic that will depend on the encoded POI and therefore can inform on when phenotypic output could be expected and temporal windows in which modifications could improve the expression of the therapeutic.

## Supplementary Material

gkaf496_Supplemental_Files

## Data Availability

Further information and requests for resources and reagents should be directed to, and will be fulfilled, by Dr Catherine Wilson (chw39@cam.ac.uk) and Dr Camilla Ascanelli (ca489@cam.ac.uk).
