# Supplementary material for: A flexible, high-throughput system for studying live mRNA translation with HiBiT technology
Source: Nucleic Acids Res. 2025 Jun 16;53(11):gkaf496. doi: 10.1093/nar/gkaf496 (PMC12168084; doi:10.1093/nar/gkaf496)
Supplement: gkaf496_Supplemental_Files [file gkaf496_supplemental_files.zip › Ascanelli & Lawrence et al 2024 NAR_Supplemental_2.pdf]

Supplemental data

A flexible, high-throughput system for studying live mRNA translation with HiBiT technology

C. Ascanelli[1†*], E. Lawrence[1†], C. A. P. Batho[1], C. H. Wilson[1*]

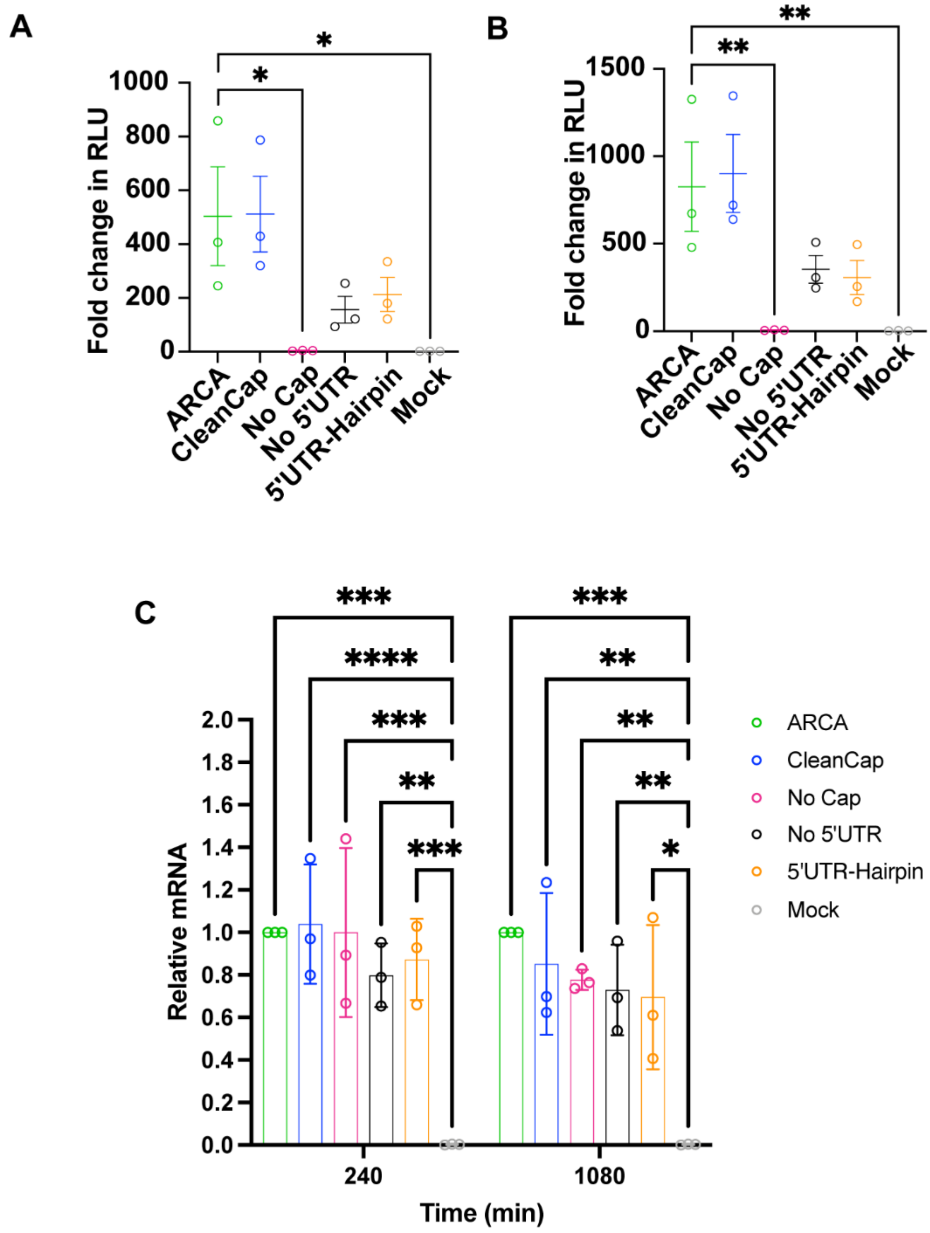

Supplemental Figure 1: Mean and SEM of Fold change in RLU from time 0 of HEK293 LgBiT cells transfected with mRNA containing different caps or 5'UTR elements are depicted at 4 hours **(A)** and 18 hours **(B)** for three biological replicates. Ordinary one-way ANOVA compared to reference sample (ARCA) was conducted. **(C)** Levels of HiBit-encoding mRNA transfected in HEK293 LgBiT cells was assessed by qRT-PCR at 4 hours or 18 hours post-transfection and normalised against reference sample (ARCA) for three biological replicates. Statistical analysis was performed using Two-way ANOVA with all samples compared within each time-points. * $p<0.05$; ** $p<0.005$; *** $0.0001<p<0.0005$; **** $p<0.0001$.

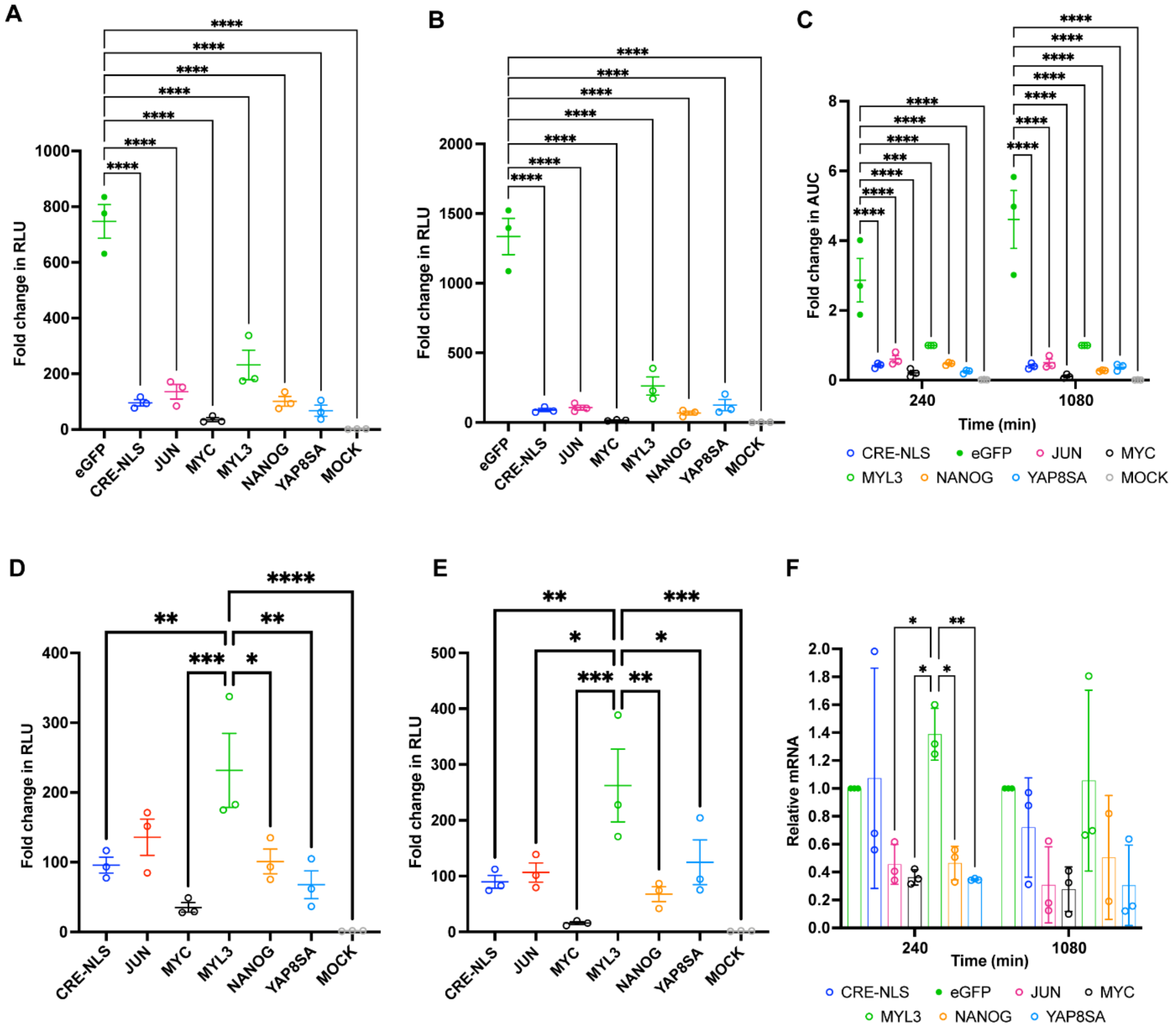

Supplemental Figure 2: Mean and SEM of three biological replicates of HEK LgBiT cells transfected with Cre-NLS-, eGFP-, Jun-, Myc-, MYL3-, NANOG-, YAP8SA-HiBiT or mock transfected. Fold change in RLU is depicted for signal at 4 hours **(A)** and 18 hours **(B)** where Ordinary one-way ANOVA was conducted comparing eGFP-HiBiT signal to all other conditions. **(C)** AUC of three biological replicates was calculated for curves up to 4 hours or 18 hours and normalised to that of MYL3 to yield fold change in AUC. Statistical analysis was conducted by Two-way ANOVA with matched values. **(D)** Mean and SEM of fold change in RLU of three biological replicates of transfected HEK LgBiT cells at 4 hours and 18 hours **(E)**; Ordinary one-way ANOVA was conducted comparing MYL3-HiBiT to all other conditions. **(F)** Levels of HiBit-encoding mRNA transfected in HEK293 LgBiT cells was assessed by qPCR at 4 hours or 18 hours post-transfection and normalised against reference sample (eGFP-HiBiT) for three biological replicates. Statistical analysis was performed using Two-way ANOVA with all samples compared within each time-points. * $p<0.05$; ** $p<0.005$; *** $0.0001<p<0.0005$; **** $p<0.0001$.

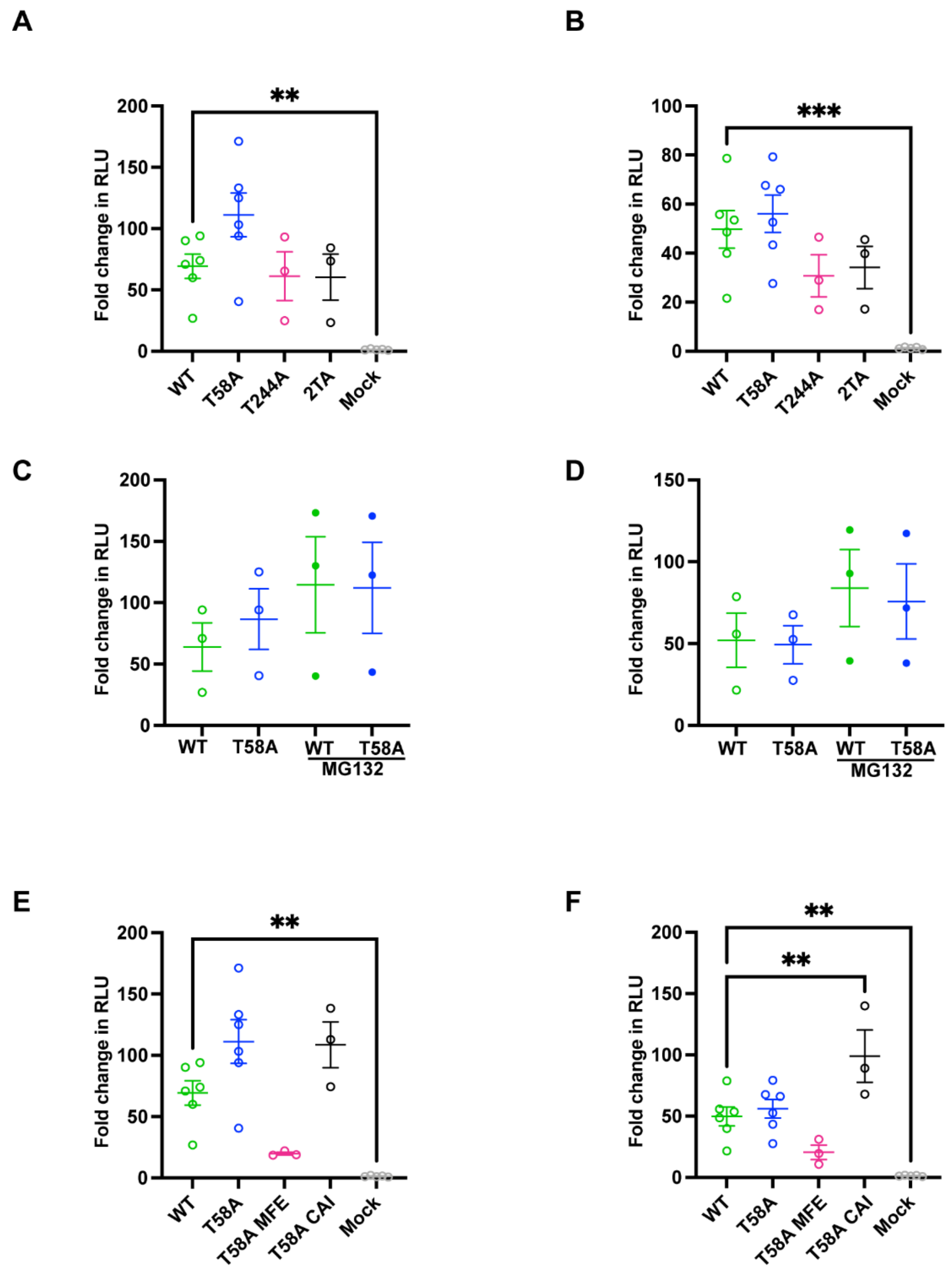

Supplemental Figure 3: Fold change in RLU of at least three biological replicates with mean and SEM depicted at 4 hours **(A)** and 18 hours **(B)** of HEK LgBiT cells transfected with Myc-HiBiT WT or stability mutants. Mean and SEM of fold change in RLU of three biological replicates of HEK LgBiT cells transfected with Myc-HiBiT WT or T58A treated with DMSO (0.005%, empty circles) or MG132 (Cf= 4μM, full circles) at 4 hours **(C)** or 18 hours **(D)**. Fold change in RLU of at least three biological replicates with mean and SEM depicted at 4 hours **(E)** and 18 hours **(F)** of HEK LgBiT cells transfected with Myc-HiBiT WT, T58A or codon optimised T58A. In all graphs, statistical analysis was performed by Ordinary one-way ANOVA with comparison to reference WT. ** $p<0.005$. *** $0.0001<p<0.0005$. WT = Wild-type Myc (green); T58A = Threonine-to-Alanine substitution at T58 of Myc (blue), T244A = Threonine-to-Alanine substitution at T244 (magenta in A,B); 2TA = Threonine-to-Alanine substitution at T58 and T244 (black in A,B); T58A MFE = Minimum-free-energy optimisation of T58A Myc (magenta in E,F), T58A CAI = Codon-adaptation-index optimisation of T58A Myc (black in E,F).

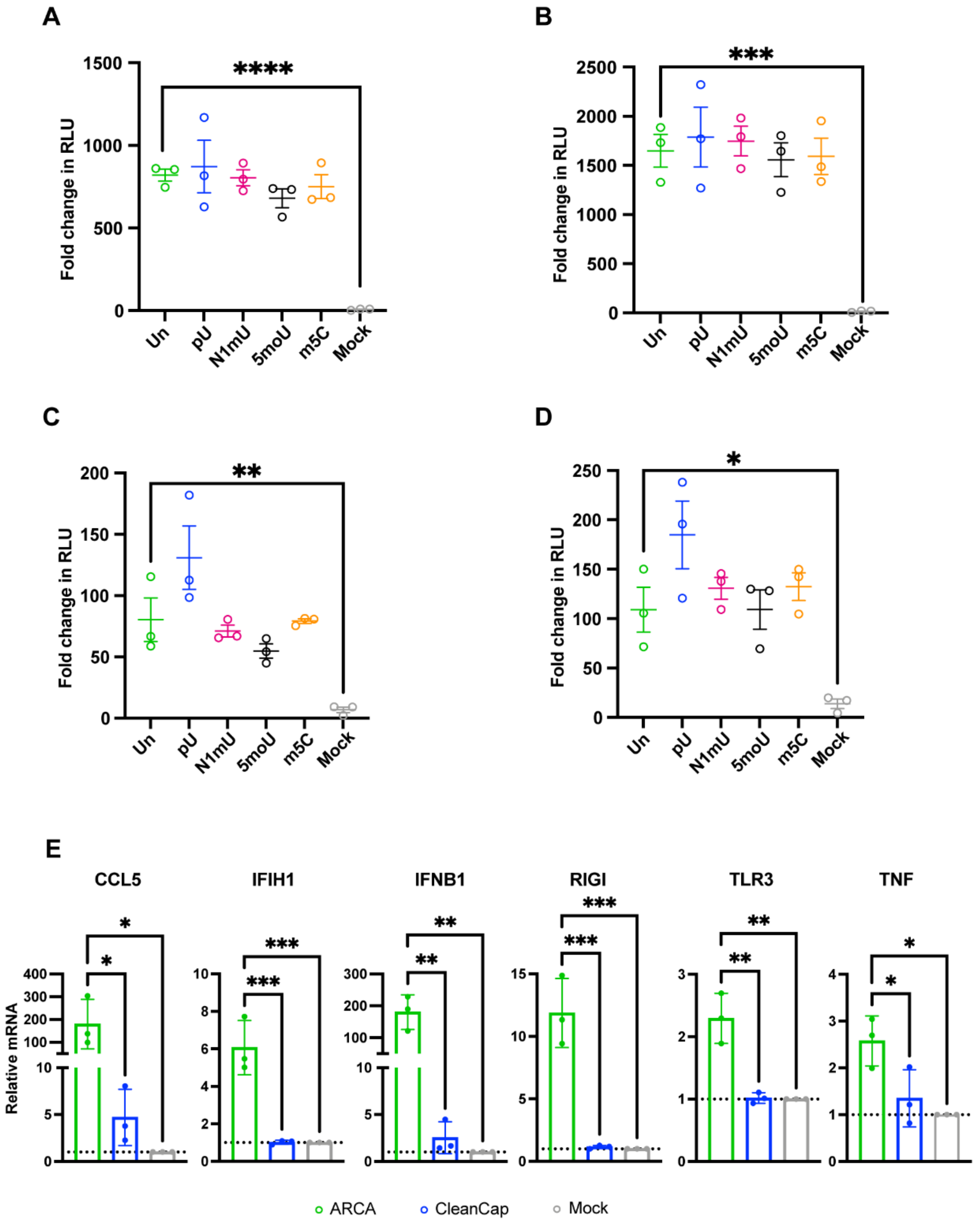

Supplemental Figure 4: Fold change in RLU of at least three biological replicates with mean and SEM depicted at 4 hours **(A, C)** and 18 hours **(B,D)** of HEK LgBiT cells transfected with eGFP-HiBit **(A,B)** or Myc-HiBiT **(C,D)**. In all graphs, statistical analysis was performed by ordinary One-way ANOVA with comparison to reference Unmodified. **(E)** qRT-PCR data for HEK293 LgBiT cells transfected with eGFP-HiBiT mRNA capped with either ARCA or CleanCap and normalised to mock transfected and compared by ordinary One-was ANOVA with multiple comparisons. * $p<0.05$; ** $p<0.005$; *** $0.0001<p<0.0005$; **** $p<0.0001$. Un= Unmodified (green); pU = Pseudouridine-5'-Triphosphate (blue); N1mU = N1-Methylpseudouridine-5'-Triphosphate (magenta); 5moU = 5-Methoxyuridine-5'-Triphosphate (black); m5c = 5-Methylcytidine-5'-Triphosphate (orange).

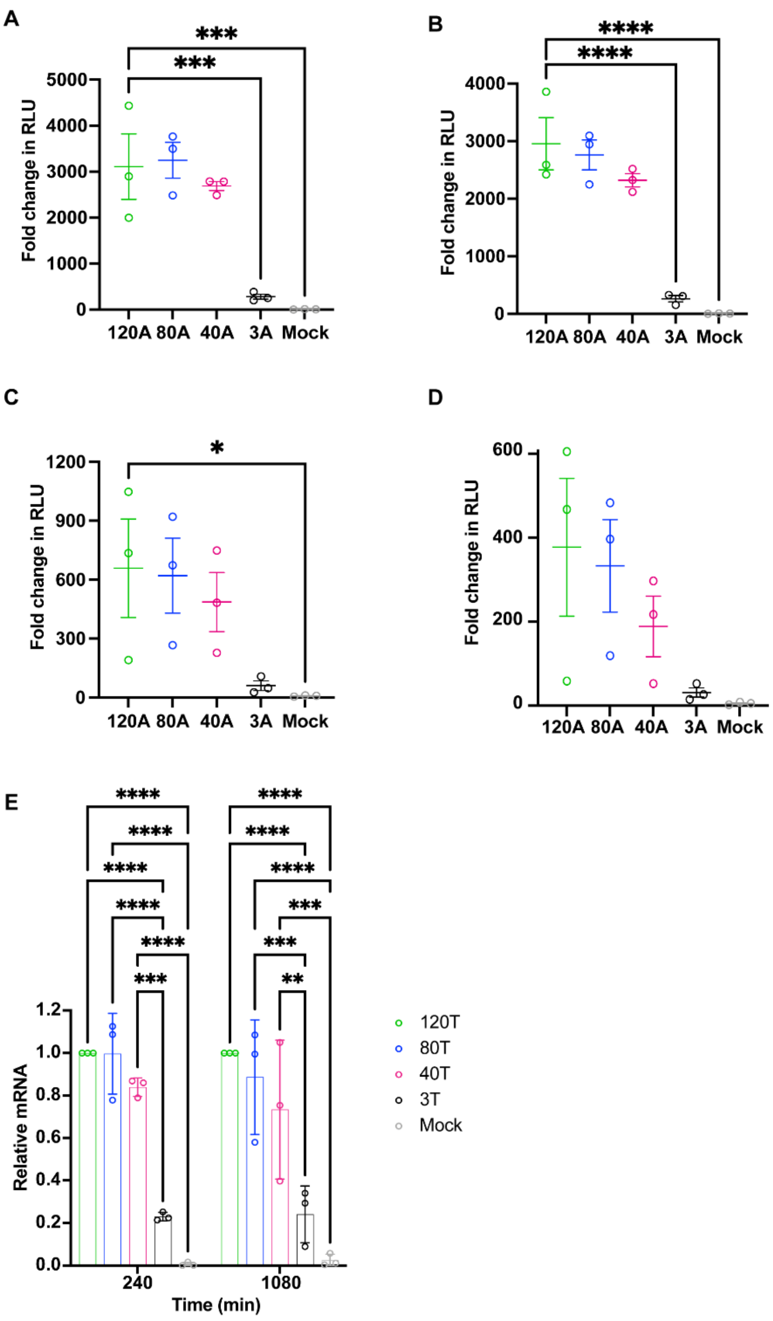

Supplemental Figure 5: Live cellular kinetic assay in HEK293 LgBiT cells transfected with eGFP-HiBiT **(A,B)** or Myc-HiBiT **(C,D)** with differing poly(A) lengths or Mock. RLU per sample was normalised to time 0 to produce fold change in RLU; mean and SEM at 4 hours **(A,C)** and 18 hours **(B,D)** are shown. Ordinary one-way ANOVA compared to 120A reference sample was performed. **(E)** Levels of HiBit-encoding mRNA transfected in HEK293 LgBiT cells was assessed by qRT-PCR at 4 hours or 18 hours post-transfection and normalised against reference sample (ARCA) for three biological replicates. Statistical analysis was performed using Two-way ANOVA with all samples compared within each time-points. * $p<0.05$; ** $p<0.005$; *** $0.0001<p<0.0005$; **** $p<0.0001$.

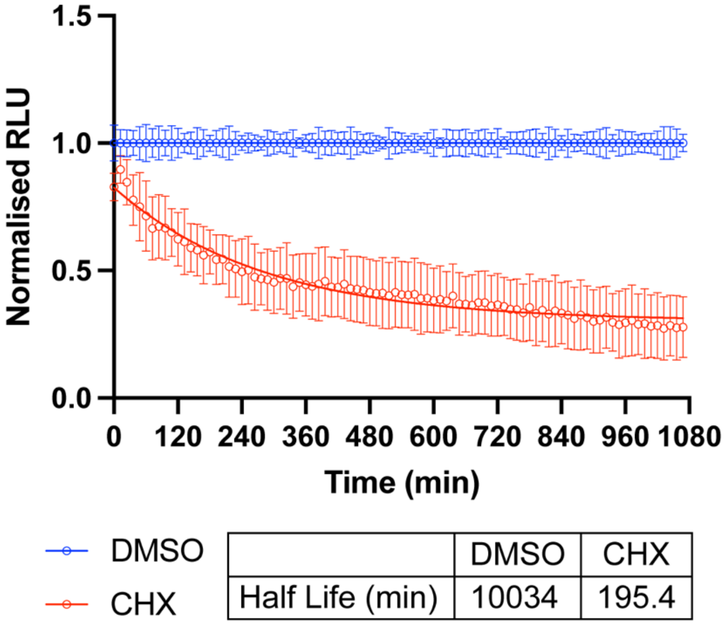

	DMSO	CHX
Half Life (min)	10034	195.4

Supplemental Figure 6: Live cellular kinetic assay in HEK293 Myc-HiBiT LgBiT cells treated with Dimethyl Sulfoxide (DMSO, Cf = 0.5%) or Cycloheximide (CHX, Cf = 100 μg/mL). A non-linear regression was applied and the half life calculated. Data shown as Mean and Standard deviation of two biological replicates.
